# Excited-state observation of active K-Ras reveals differential structural dynamics of wild-type versus oncogenic G12D and G12C mutants

**DOI:** 10.1038/s41594-023-01070-z

**Published:** 2023-08-28

**Authors:** Alexandar L. Hansen, Xinyao Xiang, Chunhua Yuan, Lei Bruschweiler-Li, Rafael Brüschweiler

**Affiliations:** 1 Campus Chemical Instrument Center, The Ohio State University, Columbus, OH USA; 2 Department of Chemistry and Biochemistry, The Ohio State University, Columbus, OH USA; 3 Department of Biological Chemistry and Pharmacology, The Ohio State University, Columbus, OH USA

**Keywords:** Molecular biophysics, Solution-state NMR, Cancer, Biophysics, Solution-state NMR

## Abstract

Despite the prominent role of the K-Ras protein in many different types of human cancer, major gaps in atomic-level information severely limit our understanding of its functions in health and disease. Here, we report the quantitative backbone structural dynamics of K-Ras by solution nuclear magnetic resonance spectroscopy of the active state of wild-type K-Ras bound to guanosine triphosphate (GTP) nucleotide and two of its oncogenic P-loop mutants, G12D and G12C, using a new nanoparticle-assisted spin relaxation method, relaxation dispersion and chemical exchange saturation transfer experiments covering the entire range of timescales from picoseconds to milliseconds. Our combined experiments allow detection and analysis of the functionally critical Switch I and Switch II regions, which have previously remained largely unobservable by X-ray crystallography and nuclear magnetic resonance spectroscopy. Our data reveal cooperative transitions of K-Ras·GTP to a highly dynamic excited state that closely resembles the partially disordered K-Ras·GDP state. These results advance our understanding of differential GTPase activities and signaling properties of the wild type versus mutants and may thus guide new strategies for the development of therapeutics.

## Abstract (web summary)

Here the authors describe the backbone structural dynamics of K-Ras in its active state by solution NMR. Comparing wild-type K-Ras to oncogenic mutants unveils cooperative transitions to a highly dynamic excited state that advances our understanding of the GTPase activities of K-Ras.

## Main

Ras proteins belong to a class of GTPase enzymes with a central role in the early stages of protein signal transduction, regulating cell growth, division and differentiation^1^. In its active form, Ras is bound to GTP, whereas in its inactive state it is bound to nucleotide guanosine diphosphate (GDP). Ras enzymatically converts GTP to GDP, a process that is accelerated in the presence of GTPase-activating proteins (GAP). Ras genes have been identified as the most frequently mutated oncogenes in human cancers, with Ras mutations associated with 19% of all cancers diagnosed in the United States and found in 3.4 million cases globally. Furthermore, as 75% of all Ras-associated cancer mutations occur in K-Ras, K-Ras has become the primary focus of Ras cancer research^2^.

**Fig. 1:**
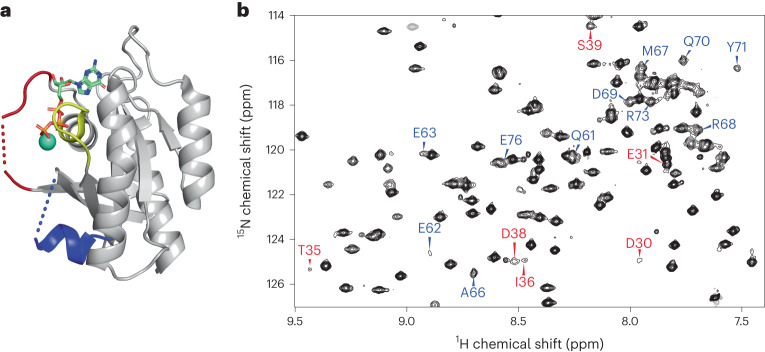
Depiction of the X-ray crystal structure of the GTP form of WT K-Ras and a representative solution NMR amide spectrum. **a**, X-ray crystal structure of WT K-Ras·GTP (PDB 5VQ2), where large sections of Switch I (red) and Switch II (blue) are missing. **b**, A section of the reference spectrum from the ^15^N CPMG relaxation dispersion data of WT K-Ras·GTP, highlighting some of the assignments of residues from Switch I (red) and Switch II (blue), many of which have previously been unobservable.

In recent years, X-ray crystallography has provided important information about the three-dimensional (3D) structure of K-Ras and its interactions with GDP, GTP and GTP analogs, and with several proteins including guanine nucleotide exchange factor (GEF), GAP and RAF^3^. The crystal structures also reveal the critical role of the Switch I (residues 30–38) and Switch II (residues 60–76) regions in protein–nucleotide interactions. However, although there is a single structure available of the wild-type (WT) K-Ras in an active GTP-bound conformation, most of the Switch regions are missing (Fig. 1a)^4^. Nuclear magnetic resonance (NMR) spectroscopy results indicate that the homolog H-Ras bound to the nonhydrolyzable GTP analog GppNHp dynamically populates multiple protein substates. Early studies using ^31^P NMR of the nucleotide revealed two states, termed states 1 and 2, slowly exchanging on the NMR chemical shift timescale^5^. State 2 is considered to be competent for downstream binding to effector proteins, and the equilibrium between the two states is shifted in favor of state 2 when K-Ras is bound to GTP or GTPγS^6,7^. Ras has also been studied via direct observation of some of its backbone NMR resonances. In another study of H-Ras bound to different GTP analogs, extreme NMR line-broadening in the Switch regions suggested the presence of conformational dynamics^8^. A subsequent ^15^N NMR Carr–Purcell–Meiboom-Gill (CPMG) relaxation dispersion analysis of H-Ras–GppNHp showed that the dynamics were distributed over different protein regions, although the properties of the Switch regions could not be studied owing to broadening of their resonances beyond detection^9^. Despite the missing Switch regions, a ^15^N chemical exchange saturation transfer (CEST) analysis of H-Ras provided the two substate populations and found large differences depending on whether native GTP or GTP analogs were used^10^. For GTP-bound WT K-Ras and the G12C and G12D mutants, around 80% of the backbone resonances were assigned recently, but the entire Switch II and a substantial number of resonances of Switch I were still missing^11^. As multidimensional NMR applications of Ras when bound to native GTP are impeded by the real-time hydrolysis of GTP, the addition of GEF was found to significantly extend the lifetime of H-Ras, allowing dynamics measurements of a larger number of residues, including several residues of the Switch regions^12^. A subsequent combined X-ray crystallography and one-dimensional ^1^H solution NMR study of WT K-Ras bound to GppCH_2_p found a significantly increased state 1 population compared with that of H-Ras, whereas the K-Ras G12D mutant favored state 2 (ref. ^13^). Together, these studies demonstrate that K-Ras behaves differently to H-Ras, with key properties of members of the Ras family being very sensitive to mutations^10^. It is therefore important to characterize the structural properties of K-Ras quantitatively and inclusively in its native GTP-bound context to provide a basis for understanding its enzymatic and signaling properties and the differences between the WT form and oncogenic mutants.

We report here backbone assignments along with comprehensive dynamics analysis of GTP-bound and GDP-bound forms of human WT K-Ras4B (residues 1–169) and its oncogenic mutants G12C and G12D, henceforth referred to as K-Ras, including the previously unobservable Switch I and Switch II residues. Experimental conditions for K-Ras·GTP were optimized to make it sufficiently stable over the time course of multidimensional NMR experiments for assignment and dynamics studies. This permitted essentially complete resonance backbone assignments of WT K-Ras·GTP and its G12C and G12D mutants, including the previously elusive yet functionally critical Switch I and II regions. Based on these assignments, the structural dynamics of K-Ras·GTP from picoseconds to milliseconds could be studied at a previously unobtained level of detail, using advanced NMR methods that provide unique insights into the function and the free-energy landscape of this system. These results reveal highly distinctive dynamic signatures for WT and mutant K-Ras·GTP and K-Ras·GDP.

## Results

### Sample preparation and resonance assignments

Despite years of NMR-based K-Ras research, many residues, including some in the key Switch I and II regions, could not be detected, and hence could not be assigned, seriously impeding the structural and dynamic characterization of this protein at atomic detail in solution. By improving the sample preparation and NMR measurement protocols (Methods), we detected and established essentially complete (>98%) backbone resonance assignments of GTP-bound WT K-Ras and its G12D and G12C mutants at room temperature (298 K). This is illustrated in the 2D ^15^N–^1^H heteronuclear single quantum coherence (HSQC) NMR spectrum (Fig. 1b) of WT K-Ras, which shows previously unobservable resonances in Switch I (red) and Switch II (blue). Although some of these peaks were significantly weaker than others or affected by peak overlap, such as D30, Y32, D33, T35, I36, E37 and D38 (Switch I) and G60, E62, E63, Q70, Y71 and E76 (Switch II), they were amenable to quantitative dynamics analysis. These advances were made possible by the optimized sample-preparation protocol, shortened NMR time using nonuniform sampling and the high sensitivity afforded by measurements at 850 MHz with a TCI cryoprobe. Notably, these results were obtained for the intact K-Ras enzyme in the presence of its native GTP substrate, with slow hydrolysis of GTP to GDP taking place during the course of the NMR experiment. To prevent *t*_1_-noise spectral artifacts due to enzymatic turnover changing sample composition, the order of the acquisition of increments along the indirect *t*_1_ dimension was randomized and interleaved with the number of scans while making use of minimal phase cycles. The backbone resonance assignments are complete to >98% (the few unassigned residues are listed in Supplementary Table 2). The availability of complete sets of resonances with high spectral quality allowed us to gain previously inaccessible, quantitative insights into the structural dynamic properties of K-Ras and its mutants.

### NMR spin relaxation

**Fig. 2:**
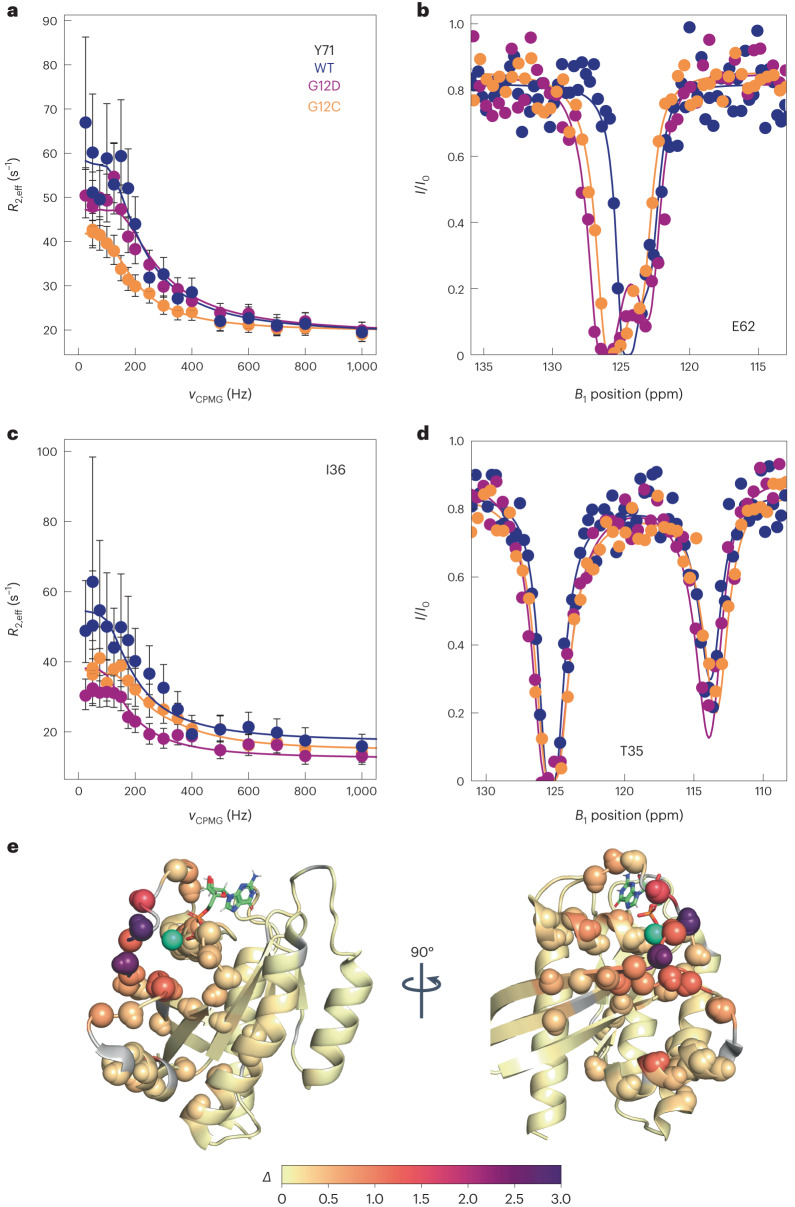
Selected ^15^N-NMR relaxation dispersion curves and ^15^N CEST profiles for K-Ras·GTP with results color-coded on 3D structure of K-Ras. ^15^N-dispersion and CEST profiles are shown for WT (dark blue), G12D (purple) and G12C (orange) K-Ras bound to GTP. **a**, Selected Switch II ^15^N CPMG dispersion. Values of *R*_2,eff_ were calculated as described in the Supplementary Information with errors derived from error propagation of the experimental uncertainties in signal amplitudes. Data are presented as the measured value plus or minus one standard deviation. **b**, Selected Switch II ^15^N CEST profiles. **c**, Selected Switch I ^15^N CPMG dispersions, presented as described in **a**. **d**, Selected Switch I ^15^N CEST profiles. **e**, Combined excited-state chemical shift differences *Δ* for WT K-Ras·GTP plotted on the K-Ras·GDP crystal structure (PDB 4OBE) for all residues, where $\varDelta =\sqrt{{(\Delta {\omega }_{\mathrm{N}}/{\sigma }_{\mathrm{N}})}^{2}+{(\Delta {\omega }_{\mathrm{HN}}/{\sigma }_{\mathrm{HN}})}^{2}}$, and *σ*_N_ and *σ*_HN_ are the standard deviations of amide ^15^N and ^1^H^N^ chemical shifts with values 5.218 ppm and 0.634 ppm, respectively. Residues with *Δ* > 0.2 are shown as spheres, whereas unobserved residues are shown in gray. The teal sphere is the Mg^2+^ ion observed in the crystal structure. Source data

Backbone ^15^N NMR spin relaxation experiments report on conformational dynamics of proteins over a large range of motional timescales. Here, we examined dynamics on (1) the microsecond to millisecond processes that are probed by CPMG and CEST experiments^14,15^ and (2) the picosecond-to-microsecond dynamics made accessible by nanoparticle-assisted spin relaxation (NASR)^16^ and traditional model-free analysis^17^. Figure 2 shows representative ^15^N CPMG relaxation dispersion and CEST saturation profiles of residues T35, I36 (Switch I), and E62 and Y71 (Switch II). Although these previously unobservable residues gave rise to some of the weakest ^15^N–^1^H HSQC cross-peaks (Fig. 1b), they could be unambiguously assigned and fully quantitatively analyzed in both CPMG and CEST experiments, as can be seen by the small error bars obtained for repeat experiments. The WT, G12C and G12D K-Ras displayed different degrees of relaxation dispersion, as shown in Fig. 2a, reflecting differences in the substate populations, differences in the chemical shift changes between the ground state and excited state, and differences in the interconversion rate constants (*k*_ex_). High-quality ^15^N and ^1^H CPMG relaxation dispersion data and ^15^N CEST profiles were measured for all three K-Ras variants with 22 to 50 (nonproline) residues showing significant ^15^N exchange effects (*R*_ex_ > 5 s^−1^, Table 1). The data were subsequently fitted to numerical expressions of conformational exchange using ChemEx^18^ software. Quantitative interpretation of the raw data was achieved with a global two-state exchange process parametrized by an exchange rate constant *k*_ex_ = *k*_21_ + *k*_12_ between the two dynamically interconverting substates 2 and 1 with populations *p*_2_, *p*_1_ = 1 – *p*_2_, and residue-specific chemical exchange differences Δϖ (Table 1).

**Table 1:** Summary of NMR-based dynamics results for WT, G12C and G12D K-Ras bound to GTP at 298 K. Uncertainties in the parameters were determined through bootstrap analysis

Sample		^15^N CPMG^a^	^1^H^N^ CPMG^a^	^15^N CEST^a^	*k*_21_ (s^−1^)	*k*_12_ (s^−1^)	*k*_ex_ (s^−1^)^b^	*p*_1_ (%)^c^
GTP	WT	29	22	28	40.6 ± 2.2	359 ± 11	400 ± 12	10.15 ± 0.47
	G12C	31	–	31	22.7 ± 0.9	303 ± 10	326 ± 11	6.97 ± 0.17
	G12D	50	22	50	27.1 ± 2.0	274 ± 16	301 ± 17	9.00 ± 0.44

^a^Total number of residues with significant conformational exchange contributions that were included in parametrization of two-site exchange model by global nonlinear least squares fitting.

^b^Globally fitted exchange rate constant *k*_ex_ = *k*_21_ + *k*_12_ using a two-state conformational exchange model consisting of a ground state (state 2) and an excited state (state 1).

^c^Globally fitted population *p*_1_ of the excited state (state 1), whereby *p*_1_ = 1 – *p*_2_.

At 298 K, conformational exchange of K-Ras·GTP followed an excellent approximation of a two-site exchange process for all three variants with the global kinetic and thermodynamic parameters depending sensitively on the residue type in position 12 (Table 1). On average, exchange proceeded at a moderately slow rate with a relatively large population of the excited state 1; the corresponding parameter values for WT were *k*_ex_ = 400 s^−1^ and *p*_1_ = 10%. Both the G12D and G12C mutants had lower values for both parameters; the G12D mutant had the slowest exchange rate of 301 s^−1^, and G12C had the lowest excited-state population (*p*_1_) of 7%.

### Chemical shifts of the excited state

**Fig. 3:**
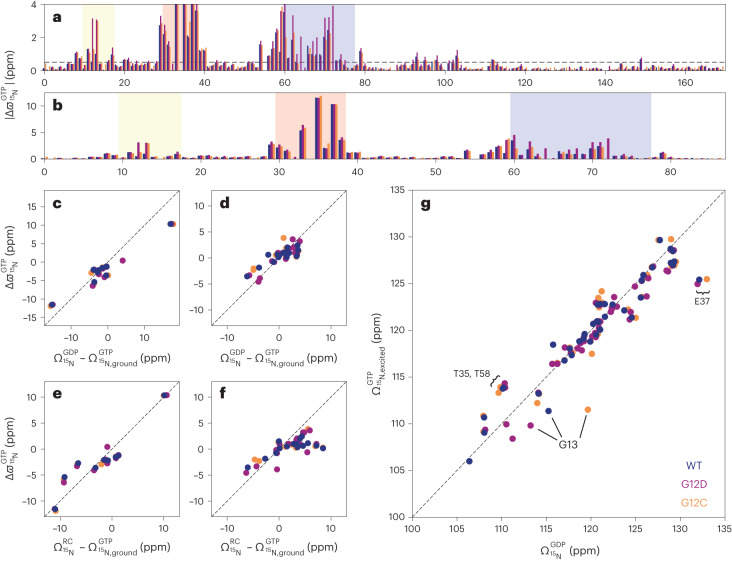
Relating ^15^N NMR chemical shifts of excited state of K-Ras·GTP to alternative states of K-Ras. In all panels, data for K-Ras·GTP for WT, G12D and G12C are colored dark blue, purple and orange, respectively. **a**, Unsigned ^15^N dynamic chemical shift differences |Δϖ| between excited and ground states, obtained from CPMG and CEST experiments (Methods), plotted against the primary sequence. The dashed line is at 0.5 ppm, and the P-loop, Switch I and Switch II regions are highlighted in yellow, red and blue, respectively. **b**, The same results are shown as in **a**, but showing the effector lobe residues only. **c**,**d**, Signed ^15^N Δϖ values for Switch I (**c**) and Switch II (**d**) residues correlated with the equilibrium chemical shifts (Ω) and their differences observed between the ^15^N–^1^H HSQC spectra of K-Ras·GDP and K-Ras·GTP, respectively. **e**,**f**, The corresponding correlations of Switch I (**e**) and Switch II (**f**) ^15^N Δϖ values with the differences between chemical shifts Ω predicted for random coil states and those observed for K-Ras·GTP. **g**, Depiction of the correlations between chemical shifts of the excited states of K-Ras·GTP and K-Ras·GDP for residues 1–86 with ^15^N |Δϖ| > 0.5 ppm. Dashed lines in **c**–**g** correspond to the diagonal with slope 1. The root-mean-square deviation and Pearson *R*^2^ correlation coefficients are provided in Table 2. Errors in the measurements are smaller than the symbol sizes. Source data

The exchange rates *k*_ex_ fell in a regime on the NMR timescale that allowed the quantitative extraction of site-specific ^15^N chemical shift changes Δϖ between the ground state 2 and excited state 1 depicted in Fig. 3a,b. The largest chemical shift changes were observed for residues 29–38 (V29, D30, E31, D33, T35, I36, E37, D38) directly preceding or residing in Switch I, and for residues 54–72 (D54, L56, D57, T58, A59, G60, E62, Y64, A66, M67, R68, D69, Q70, Y71, M72) and L79 immediately preceding or residing in Switch II; these findings support the long-held notion that both Switch regions have a key role in functionally important conformational dynamics processes of K-Ras. In addition, significant chemical shift changes were observed for V8, V9, A11, G12X, G13 and S17, which are either part of or immediately precede the P-loop. The vast majority of changes occurred in the amino-terminal effector lobe (residues 1–86), whereas in the carboxy-terminal half of K-Ras (residues 87–169) changes also occurred but were overall much smaller and more scattered across the primary sequence (Supplementary Fig. 1).

The conformation or conformational ensemble of an excited state is difficult to determine based on backbone ^15^N and ^1^H^N^ chemical shift information alone. However, it is possible to compare the chemical shifts of the excited state with those of alternative, experimentally established states or with predicted chemical shifts to draw conclusions about their structural similarity (Fig. 3c–g). Such a comparison is depicted in Fig. 3g between the ^15^N chemical shifts of the excited state of all residues of K-Ras·GTP and the equilibrium chemical shifts of K-Ras·GDP; this yielded close agreement, with a high Pearson *R*^2^ correlation of 0.88. When this comparison was limited to signed Δϖ values observed for residues that belonged to either Switch I (Fig. 3c) or Switch II (Fig. 3d), the *R*^2^ values were 0.95 and 0.69, respectively (Table 2). Using an alternative random coil model for Switch I and II (Fig. 3e,f), with random coil chemical shifts predicted based on the amino acid sequence using the POTENCI^19^ software, resulted in reduced *R*^2^ correlations of 0.88 and 0.47. These results show that the excited state of K-Ras·GTP adopts a state that resembles K-Ras·GDP, with a degree of flexibility for parts of Switch I and Switch II similar to that of a random coil conformation. Residues that deviated most from the K-Ras·GDP model (Fig. 3g) were those that were closest to the γ-phosphate of GTP and therefore experienced additional chemical shift changes that were probably caused by the change in chemistry between GTP and GDP rather than structural dynamics (Supplementary Fig. 2).

**Table 2:** Summary of the excited-state chemical shift correlations shown in Fig. 3

	Switch I: 29–37		Switch II: 59–78		Effector lobe: 1–86
Abscissa	$\Omega^{\mathrm {RC}}_{15{\mathrm N}}-\Omega^{\mathrm {GTP}}_{15{\mathrm {N,ground}}}$	$\Omega^{\mathrm {GDP}}_{15{\mathrm N}}-\Omega^{\mathrm {GTP}}_{15{\mathrm {N,ground}}}$	$\Omega^{\mathrm {RC}}_{15{\mathrm N}}-\Omega^{\mathrm {GTP}}_{15{\mathrm {N,ground}}}$	$\Omega^{\mathrm {GDP}}_{15{\mathrm N}}-\Omega^{\mathrm {GTP}}_{15{\mathrm {N,ground}}}$	$\Omega^{\mathrm {GDP}}_{15{\mathrm N}}$
Root-mean-square deviation	2.10 ppm	3.05 ppm	3.39 ppm	1.56 ppm	2.15 ppm
*R*^2^	0.875	0.951	0.473	0.686	0.881

Ω stands for equilibrium chemical shifts.

### Nanoparticle-assisted spin relaxation

**Fig. 4:**
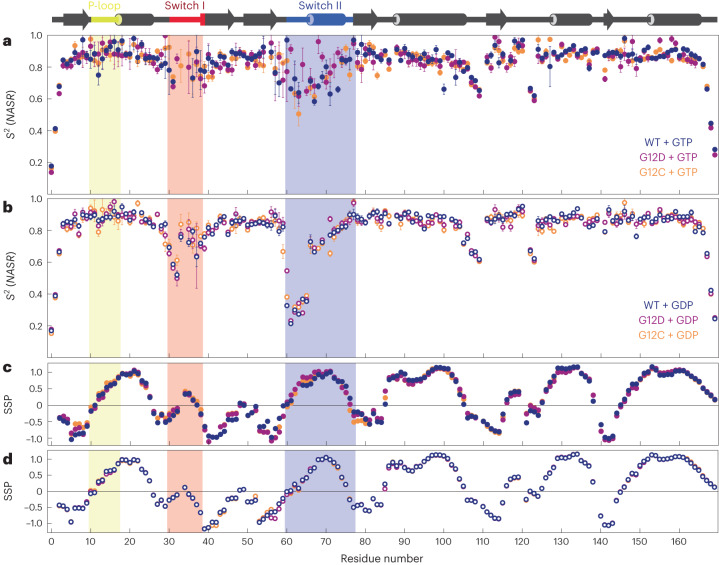
Backbone dynamics of K-Ras·GTP (filled circles) and K-Ras·GDP (open circles) for WT (dark blue) and G12D (purple) and G12C (orange) mutants on the picosecond-to-microsecond timescale and SSP. Secondary structural elements are shown at the top of the figure, with the P-loop, Switch I and Switch II regions shaded light yellow, red and blue, respectively. **a**,**b**, Backbone N–H *S*^2^ order parameters were determined by the NASR approach for K-Ras·GTP (**a**) and K-Ras·GDP (**b**). Data are presented as best-fit values plus or minus one standard deviation. Errors for the data points were determined through Monte Carlo simulation and standard error propagation. **c**,**d**, SSP of each variant derived from K-Ras·GTP (**c**) and K-Ras·GDP (**d**) ^13^Cα and ^13^Cβ chemical shifts. Source data

Relaxation dispersion and CEST data reflect conformational exchange on the millisecond timescale, whereas ^15^N-*R*_1_ and *R*_2_ relaxation parameters provide information about additional dynamic processes of N–H bond vectors occurring on faster timescales. We used the NASR method, which measures the change in transverse *R*_2_ relaxation in the presence and absence of silica nanoparticles, to report directly on picosecond-to-microsecond motions^16^. The extracted *S*^2^(NASR) order parameters are shown in Fig. 4a,b for the three variants of K-Ras·GTP and K-Ras·GDP. For all forms, regular secondary structures were internally rigid, as reflected by high *S*^2^(NASR) order parameters, whereas the N and C termini had increased mobility (low *S*^2^(NASR)), as is typical for many proteins. In addition, loop residues E107–V109 and S122–R123, which are located in the C-terminal half of K-Ras, exhibited increased mobility across all forms. In K-Ras·GTP, moderately increased mobility was found for Switch II residues, with several residues having *S*^2^(NASR) < 0.65, whereas Switch I residues were motionally restricted with *S*^2^(NASR) > 0.68. For K-Ras·GDP, the NASR profiles changed significantly, showing increased mobility in Switch I, especially for E31 and Y32, with *S*^2^(NASR) values between 0.50 and 0.61; there were even larger amplitude motions for Switch II residues G60–S65, with *S*^2^(NASR) values between 0.21 and 0.37 for the WT. The NASR profiles of the mutants closely resembled those of the WT except for G12D, in which residue G60 of K-Ras·GDP was significantly more rigid (*S*^2^(NASR) = 0.55) than in the WT and G12C (*S*^2^(NASR) = 0.33–0.38). With respect to A59, G12C was more flexible than the WT and G12D (*S*^2^(NASR) = 0.67 versus 0.82 and 0.85).

The secondary structure propensities^20^ (SSP) of all three variants for the GDP-bound and GTP-bound forms are shown in Fig. 4c,d. The largest differences between the GDP-bound and GTP-bound forms occurred in Switch I and Switch II. In K-Ras·GDP, Switch I residues P34 and T35 had SSP indices close to zero, consistent with a high degree of intrinsic disorder, whereas in K-Ras·GTP the same residues had values of about 0.32–0.42, indicative of a more structured state. Similarly, Switch II residues E62–A66 had systematically smaller (absolute) values in K-Ras·GDP than in K-Ras·GTP, suggesting that this section of Switch II is overall significantly more disordered in the GDP-bound form. From residue M67 onward, Switch II becomes better structured, with the apex of the SSP index approaching 1 around residue 70 for both nucleotide ligands. This interpretation is consistent with the NASR dynamics results and closely mirrors results obtained with TALOS-N^21^ software (Supplementary Fig. 3).

## Discussion

### State 2 versus state 1 of K-Ras

Since the discovery by ^31^P NMR^5^ that K-Ras·GTP populates an alternative state 1 distinct from its major state 2, there has been intense interest in the biological roles and structural properties of the two states. Variations of the equilibrium constant between the two interconverting states for different small GTPases and their interactions with effector proteins have been associated with different biochemical properties^22–25^. In particular, state 1 promotes nucleotide exchange while inhibiting interactions with downstream effector proteins, whereas state 2 allows effector binding and GTP hydrolysis. States 1 and 2 were subsequently structurally characterized by X-ray crystallography of selected K-Ras mutants bound to GDP or GTP analogs^26,27^, but detailed structural dynamic information for K-Ras bound to the native GTP ligand in solution remained elusive. Such information is critical, as the crystal structures do not necessarily reflect the substates present in solution. Based on the equilibrium constants^6^ between state 2 and state 1, we assigned the dominant ground state observed in our CPMG and CEST experiments to state 2 and the excited state to state 1. This is further supported by the structural dynamic characteristics of the ground versus excited state in the context of the known functional properties of states 1 and 2 described below.

Although the Switch I and II regions of K-Ras have been known to make critical contacts with the GTP substrate and are important for GTPase activity, they have remained largely undetectable by X-ray crystallography and solution NMR. By optimizing NMR samples and experimental conditions, we have been able to detect and assign essentially all backbone chemical shifts of both Switch I and II for K-Ras·GTP WT, G12D and G12C. Specifically, 100% of nonproline residues could be assigned for G12D and 98% for WT (missing assignments: Y64, S65, M72) and G12C (missing assignments: Q61, Y64, M72) (Supplementary Table 2). This allowed the quantitative capture of the dynamics of previously unobservable residues in Switch I and II.

### Global 2-state exchange and free-energy diagram of K-Ras·GTP and its mutants

**Fig. 5:**
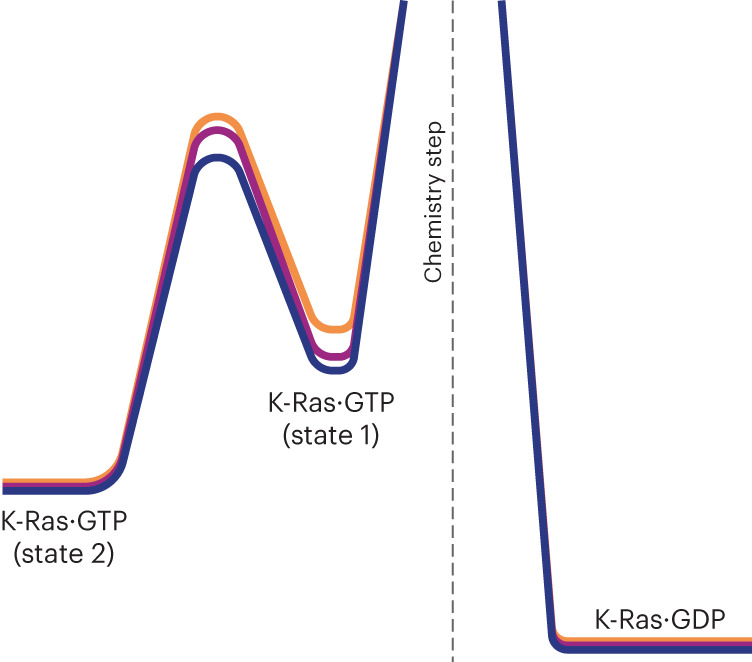
Free energy diagram of the enyzmatic reaction of K-Ras. Free-energy scheme of K-Ras along its GTPase reaction coordinate for the WT (dark blue) in comparison with the oncogenic G12D (purple) and G12C (orange) mutants.

CPMG and CEST data sensitively report on conformational exchange on the biologically significant millisecond timescale, allowing screening for one or several transiently populated alternative conformational states that are in dynamic equilibrium with the major state. CEST experiments are complimentary to CPMG, as they directly depict both the magnitude and the sign of the chemical shift of the excited state, that is, whether it is up-field or down-field shifted relative to the ground state. This is important when modeling the structure of the excited state with alternative structural states, as discussed below. The CEST data for K-Ras·GTP unambiguously show the existence of a single excited state, which is manifested by the presence of a second dip in the CEST profiles of a sizable number of residues, as illustrated in Fig. 2 for T35 in Switch I and E62 in Switch II. Even for E62, which gave rise to the weakest cross-peak in the entire HSQC spectrum (lower left corner of Fig. 1b), the presence of an excited state of this residue was evident for all three K-Ras·GTP variants (Fig. 2d). Within the NMR detection limits, there was no indication that any residue substantially populates more than one excited state on the microsecond to millisecond timescale. The CPMG and CEST data for all three K-Ras·GTP variants could be fitted to global two-state exchange processes for the WT and both mutants; the best-fitting model parameters are listed in Table 1. K-Ras·GTP undergoes thermally activated, stochastic transitions between a dominant conformational state (ground state) and an alternative conformational state (excited state) cooperatively involving Switch I, Switch II, the P-loop and a few other regions discussed further below. WT K-Ras shows distinct behavior, with both *k*_21_ = 40.6 s^−1^ and *p*_1_ = 10.2% elevated compared with those of the oncogenic mutants G12D (*k*_21_ = 27.1 s^−1^ and *p*_1_ = 9.0%) and G12C (*k*_21_ = 22.7 s^−1^ and *p*_1_ = 7.0%). Hence, WT K-Ras has an excited state that is more accessible both thermodynamically (larger *p*_1_) and kinetically (larger *k*_21_) than those of the oncogenic mutants; this difference may be instrumental in the reduced GTPase activity of mutant K-Ras (vide infra). The corresponding free-energy diagram of the three K-Ras variants (Fig. 5) highlights distinct differences in populations and the free energy of the transition state of the WT versus mutants. In contrast to K-Ras·GTP, we found no experimental evidence that K-Ras·GDP undergoes conformational exchange on the millisecond timescale with a significantly populated excited state (Supplementary Fig. 4).

The residues participating in conformational exchange of K-Ras·GTP were located predominantly in the N-terminal effector lobe (Fig. 3a,b). These residues are V8, V9, A11, G12X and G13 in the P-loop; residues V29–Y40, which essentially represent the entire Switch I; residues D54–M72, constituting a large part of Switch II; and L79 and C80 of the β4-strand. G12X and G13 displayed notable differences in Δϖ among the WT and mutants, whereas for most other residues the WT and the two mutants had similar Δϖ. This is not surprising, as G12X is the mutation site; hence, residues in this region experience a different chemical environment that is reflected in the chemical shifts of the ground state and the excited states and their differences. Residues 92–98, belonging to the C-terminal end of the α3-helix in the C-terminal allosteric lobe (residues 87–169), displayed somewhat more modest exchange-induced chemical shift modulations. As the P-loop is wedged between residues 92–98 and L79/C80 of the β4-strand on one side and the GTP ligand on the other, the dynamic modulation of the P-loop during exchange requires adjustments in the local structure and environment of the α3-helix and β4-strand residues, which were manifested in the observed chemical shift changes. These latter regions of the α3-helix and β4-strand may represent allosteric sites; hence, it may be possible to modulate K-Ras signaling by targeting them with ligands that stabilize the excited state (state 1), thereby disrupting interactions of K-Ras·GTP with downstream effector proteins.

### Intrinsic differences between K-Ras state 2 and state 1 structure and dynamics

As well as providing global exchange dynamics parameters, the CPMG and CEST experiments also return residue-specific chemical shift differences (Δϖ) between the two states, thereby shedding light on the structure of the excited state. A direct way of annotating Δϖ values involves comparing them with chemical shifts that are known or expected for alternative states. Here, the best agreement was found when using the conformational ensemble of K-Ras·GDP as a model for the excited state, with Switch I behaving as random coil (Fig. 3). This is consistent with the SSP data, which were close to zero for Switch I of K-Ras·GDP^28^ (indicative of random coil) (Fig. 4d), whereas for K-Ras·GTP the corresponding SSP values were clearly elevated for residues positioned toward the middle of Switch I (Fig. 4c). This conclusion is supported by the NASR *S*^2^ data for K-Ras·GDP (Fig. 4b), providing direct evidence that Switch I is substantially more flexible there than in K-Ras·GTP. These results further corroborate our experimental finding that Switch I of K-Ras·GTP undergoes a major transition between a structured ground state and a floppy excited state that behaves like K-Ras·GDP.

The SSPs of K-Ras·GDP were also close to zero for the initial part of Switch II (residues G60–S65) before starting to rise markedly from residue A66 onward. The NASR profile (Fig. 4b) shows strikingly low *S*^2^(NASR) order parameters for this initial part, with values ranging from 0.21 to 0.39, compared with the range of 0.66–0.90 for the remainder of Switch II (residues 66–76). By contrast, the NASR dynamics of K-Ras·GTP were much more constrained, with *S*^2^ values ranging between 0.6 and 0.9 (Fig. 4a). For both Switch I and Switch II, K-Ras·GDP is a suitable model of the excited state of K-Ras·GTP, suggesting a conformational exchange behavior where both Switches have limited flexibility in the ground state and significantly more heterogeneous dynamics in the excited state, with the N-terminal parts of both Switch I and II being most dynamic. Notably, only limited dynamics were observed for the two Switch regions by traditional model-free *S*^2^ order parameter analysis (Supplementary Fig. 5). This shows the extended range of dynamics information provided by NASR, indicating that the Switch dynamics take place on the 10 ns to 1 μs timescale^16^.

### Minimal X-ray structural ensemble models of K-Ras·GDP

**Fig. 6:**
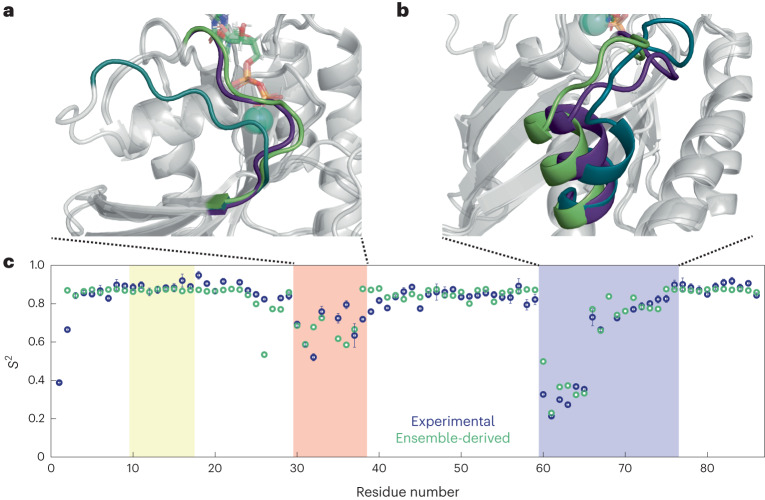
X-ray-structure-derived minimal ensemble of K-Ras·GDP Switch I and Switch II conformations and back-calculated N–H *S*^2^ order parameters. **a**,**b**, Backbone ribbon plots of K-Ras·GDP crystal structures highlighting Switch I (**a**) and Switch II (**b**). The ensemble consists of the WT K-Ras·GDP crystal structure (PDB 6MBU), the G12D mutant structure (PDB 4EPR, with engineered mutation C118S) and the A59G mutant structure (PDB 6ASE). The GDP nucleotide is shown in stick representation, and Mg^2+^ ions are shown as teal spheres. The Switch regions are indicated with nongray colors (6MBU: green, 4EPR: dark purple, 6ASE: dark cyan). **c**, Comparison between the ensemble-derived backbone N–H *S*^2^ order parameters (green) and the experimental *S*^2^(NASR) of WT K-Ras·GDP (dark blue). The *S*^2^(NASR) data are presented as the best fit plus or minus one standard deviation, as described in the caption of Fig. 4. Populations of 47% (6MBU), 39% (4EPR) and 14% (6ASE) best reproduce the experimental *S*^2^(NASR) results. The P-loop, Switch I and Switch II regions are highlighted in yellow, red and blue, respectively. The root-mean-square deviations between the ensemble-derived and experimental *S*^2^ values were 0.12 for the Switch I region and 0.06 for the Switch II region. Source data

There is no experimental structural ensemble of the dynamics observed in Switch II of K-Ras·GDP in solution. However, many X-ray crystal structures of WT K-Ras·GDP exist, with their Switch II structures differing by a variable degree from each other. With these, one can construct ensembles of interconverting crystal structures to interpret the experimental *S*^2^(NASR) profiles. In particular, the different orientations of the Switch II-α2 helix adopted by the two WT structures (PDB 4OBE ref. ^29^ and 6MBU ref. ^30^) can explain the positive *S*^2^(NASR) gradient observed between residues A66 and E76. Furthermore, the pronounced *S*^2^(NASR) minimum in the Switch II loop region requires the presence of the G12D mutant structure 4EPR^31^ in addition to the WT K-Ras·GDP conformations found in crystal structures (Supplementary Fig. 6b). For Switch I, the vast majority of the reported K-Ras·GDP X-ray crystal structures (reviewed, for instance, in ref. ^32^) adopt the same conformation, except for the D33E (PDB 6ASA) and A59G (PDB 6ASE) mutants, where 6ASA and 6ASE possess nearly identical and more extended Switch I conformations^33^. The characteristic *S*^2^(NASR) profile can be accounted for only if one assumes significant populations from at least three conformers, namely WT 6MBU, G12D mutant 4EPR and A59G mutant 6ASE, with populations 47%, 39% and 14%, respectively (Fig. 6). This is the minimal X-ray ensemble found to best reproduce the *S*^2^(NASR) profile; the introduction of the other WT structure 4OBE did not result in significant further improvement (Supplementary Fig. 6e). This ensemble closely reflects the *S*^2^(NASR) profile for the Switch II region except for G60. For Switch I, the agreement is best for residues D30, E31, D33 and E37, and the ensemble somewhat overestimates *S*^2^ for residues Y32, D38 and S39. Furthermore, it underestimates *S*^2^ for N26, where the differences between 6MBU and 6ASE at the end of the α1-helix lead to lowered *S*^2^ values, whereas *S*^2^(NASR) suggests a more rigid behavior for residues immediately preceding D30. This analysis shows how the diverse set of X-ray crystals available for K-Ras·GDP can serve as templates for interconverting conformers in solution on the submicrosecond timescale. Such structural ensembles can be further refined by molecular dynamics (MD) computer simulations using the experimental *S*^2^(NASR) data as quantitative benchmarks (see below).

### WT K-Ras·GTP is more dynamic than G12D and G12C

Our results reveal the structural nature of the two significantly populated and functionally distinct substates 1 and 2 of K-Ras·GTP in solution. Based on its backbone ^15^N chemical shifts, the excited state 1 is K-Ras·GDP-like, exhibiting high flexibility for specific portions of Switch I and II. This is in contrast to the ordered and structurally much better organized state 2, which in terms of signaling corresponds to the active state of K-Ras, as it is binding competent with respect to downstream effector proteins. The NMR results show that WT K-Ras·GTP is dynamically more active compared with the oncogenic mutants G12D and G12C, having the highest population of excited state 1 together with the highest *k*_21_ rate constant between the ground state (state 2) and the excited state 1. Together with the P-loop, Switch I and Switch II undergo the largest structural–dynamic transformations as the protein is shuttling between the two states. The dynamic activities of WT, G12C and G12D characterized here correlate with their respective GTPase turnover rates^34,35^, ranging between 4.03 × 10^−5^ s^−1^ (WT) and 1.13 × 10^−5^ s^−1^ (G12C).

### The excited state of K-Ras·GTP is highly dynamic and K-Ras·GDP-like

The correlations between the excited state of chemical shifts and the shifts for the GDP or random coil states (Fig. 3c,d,g) are high but not perfect. This is unsurprising, as K-Ras·GDP and the K-Ras·GTP excited state 1 differ chemically by the absence or presence of the γ-phosphate group of the nucleotide, which can cause significant chemical shift changes of surrounding residues without necessarily involving structural changes. Indeed, the residues that deviate the most (Fig. 3g) belong to regions in close proximity to the γ-phosphate (Supplementary Fig. 2). Taken together, our CPMG and CEST results show that the excited state of K-Ras·GTP is K-Ras·GDP-like, with Switch I adopting in good approximation a random coil state. It should be noted that although the excited state of K-Ras·GTP is highly dynamic, the *S*^2^(NASR) profile shows only slightly reduced *S*^2^ values in Switch I and II compared with the rest of the protein (Fig. 4a). This is because of the dominance of the ground state of K-Ras·GTP (*p*_2_ = 90%), which is much more ordered in both Switch regions.

These results help rationalize why the dominantly populated K-Ras·GTP ground state corresponds to state 2, whereas the excited state is state 1, along with their distinct mechanistic roles. Owing to its K-Ras·GDP-like nature, state 1 is able to mimic the known functional behavior of K-Ras·GDP in both its favorable interactions with GEF for nucleotide exchange and its biological inactivity by preventing interactions with effector proteins. By contrast, state 2 is the active state of K-Ras·GTP that interacts with effectors, enabling downstream signaling. The K-Ras mutants G12C and G12D have higher populations of state 2 versus state 1, which makes them more competent for effector interactions and further compounds their diminished GTPase activity. This amplifies the signaling activity of the mutants that is the root cause of their oncogenicity.

The observed excited-state dynamics can also help us to better understand K-Ras from an enzymatic perspective. NMR-based observations have identified the spontaneous sampling of excited-state conformations in enzymes as critical components for catalysis^36–39^. For example, for dihydrofolate reductase, excited conformers of a series of ground states along the reaction pathway were found to correspond to structures belonging to states that immediately follow in the cycle^40^; and for arginine kinase, the excited state of the Michaelis complex was found to adopt the structure of the transition-state analog of the phosphorylation reaction of the arginine substrate^41^. The similarity between the excited state 1 of K-Ras·GTP and the product of the GTPase reaction, K-Ras·GDP, follows the same pattern; *k*_21_ (40.6 s^−1^) is significantly faster than *k*_cat_, which in the absence of GAP is less than 4 × 10^−5^ s^−1^(refs. ^34,35,42^). This suggests that although it stochastically samples the product-like K-Ras·GDP state, K-Ras·GTP successfully undergoes GTP hydrolysis only once every 10^7^ transitions. This low enzymatic efficiency, which is a hallmark of K-Ras, is the reason that WT K-Ras requires the help of GAP to accelerate turnover. When interacting with GAP and effector proteins such as RAF1 (ref. ^43^), K-Ras·GTP must be in state 2 and not state 1, as binding to the GDP-bound-like state 1 would also allow binding to K-Ras·GDP, thereby abolishing the signaling selectivity of the active state.

### Synergies between NMR and MD simulations

Over the years, numerous computational studies have been performed with the goal of elucidating the functional properties of Ras proteins in relationship to experiments^44^. Early studies focused mostly on H-Ras^45–47^, but owing to its distinct behavior those findings cannot be directly transferred to K-Ras; this has also been confirmed by computation^47,48^. An extensive MD simulation study of WT K-Ras and its G12 mutants in their GDP-bound and GTP-bound forms found substantial dynamics in the Switch regions, with other protein areas sampling distinct substates, but no significant changes in dynamics were observed between WT and its mutants, nor between GTP-bound and GDP-bound states of the same mutant^49^. Hence, past simulations have been unsuccessful in revealing distinct differences in dynamics between the GTP-bound and GDP-bound states reported here.

Although MD simulations of K-Ras·GDP can start from well-defined X-ray crystal structures^50^, K-Ras·GTP represents a major challenge for MD owing to a lack of complete experimental structures as starting points. Starting structures for MD have been constructed by simply replacing GDP by GTP in a K-Ras·GDP structure^49^ and modeling in missing residues, followed by docking simulations of GTP to the structure^51^, or by using X-ray structures of the Q61H mutant bound to a GTP analog^52^. These procedures clearly introduce an amount of uncertainty in the initial structure, with consequential impact on the simulation outcome.

An equally important challenge has been the validation of the ensuing MD trajectories, especially for the functionally vital Switch regions, which have been unobservable by both NMR and crystallography. The essentially complete quantitative body of experimental data of the backbone structural dynamics of K-Ras presented here, covering both Switches, provides key benchmarks for molecular modeling, including MD, of K-Ras. It will allow the critical assessment of MD trajectories and other conformational ensembles of K-Ras and its mutants in their GTP-bound and GDP-bound states. Although *k*_ex_ between states 2 and 1 is too slow to be captured by traditional MD simulations, the site-specific CPMG/CEST-derived chemical shift information (Fig. 3) of the two states will allow critical comparisons between experimental and predicted chemical shifts^50,53^. Such information should allow the generation of more realistic conformational ensembles of K-Ras·GTP in its ground and excited states, deepening our understanding of its diverse functional behavior. These ensembles, together with the sample-preparation protocol introduced here for detection and assignment of Switch resonances, should prove powerful for future investigations, such as ligand screening toward the development of drugs that bind to specific pockets of K-Ras mutants^54^, and for studying in atomic detail the structure and dynamics of the interactions of K-Ras with GEF, GAP and a myriad of effector proteins.

## Methods

### Human K-Ras4B G-domain cloning and expression

The WT G-domain of human K-Ras4B (residues 1–169), referred to as K-Ras, was subcloned by PCR amplification of the corresponding DNA sequence from a plasmid into expression vector *pTBSG1* (ref. ^55^) and verified by Sanger sequencing. The *pTBSG1_kRaswt* plasmid was used as a template to generate *pTBSG1_kRasG12C* and *pTBSG1_kRasG12D* plasmids using a site-directed mutagenesis kit (Agilent). Sanger sequencing was subsequently used to verify the correct DNA coding sequences. All three plasmids were individually transformed into *Escherichia coli* strain BL21(DE3) for protein overexpression and uniform isotope ^15^N- or ^15^N,^13^C-labeling for NMR measurements. The oligonucleotide sequences of all *K-Ras* constructs (WT, G12C and G12D) are given in Supplementary Table 3.

Protein expression of all three forms of the K-Ras G-domain was carried out in M9 minimal media. For U-^15^N labeling, ^15^N NH_4_Cl (1 g l^−1^) was used as the sole nitrogen source, and for (U-^15^N, U-^13^C)-double labeling, ^15^N NH_4_Cl (1 g l^−1^) and ^13^C glucose (4 g l^−1^) were used as the sole nitrogen and carbon sources, respectively. Isotopes were purchased either from CIL or Isotech. *E. coli* culture was grown at 37 °C to optical density 0.7 and induced by IPTG (Fisher Scientific) overnight at 25 °C. Protein purification was performed as described previously^55^.

### NMR sample preparation

For the preparation of K-Ras·GDP samples, purified protein was buffer-exchanged with a centrifugal filter (Amicon Ultra, molecular weight cut-off of 3 kDa) in 20 mM HEPES buffer (pH 7.0), concentrated to 650–750 μM, and supplemented with 5 mM GDP (Sigma), 5 mM MgCl_2_, 5 mM BME and 5% D_2_O for NMR measurements.

For the preparation of K-Ras·GTP samples, purified protein was buffer-exchanged first in 20 mM HEPES and 15 mM EDTA buffer (pH 7.0), followed by another buffer exchange in 20 mM HEPES buffer (pH 7.0) before being concentrated. After the protein concentration had been measured, the protein solution was diluted to 100 μM with 20 mM HEPES buffer (pH 7.0), and GTP ligand (Fisher Scientific) was added to a final concentration of 10 mM for further buffer exchange, a step that was then repeated twice. The final, concentrated protein solution (650–750 μM) was supplemented with 5 mM MgCl_2_, 5 mM beta-mercaptoethanol and 5% D_2_O for NMR measurements.

### Resonance assignments

NMR spectra for the sequence-specific NMR resonance assignments were recorded on a Bruker Avance III 850 MHz spectrometer (Bruker), equipped with a 5 mm TCI triple-resonance HCN cryoprobe and *z*-axis gradient. A series of six standard triple-resonance experiments^56^ were subsequently performed using sensitivity-enhanced gradient coherence selection^57,58^, semi-constant time acquisition in the ^15^N dimension^59^ and nonuniform sampling following a Poisson-gap sampling schedule^60^. In addition, 3D ^15^N-edited NOESY and 3D CNH-NOESY^61^ were performed using uniform sampling with a mixing time of 180 ms. Full details are provided in the Supplementary Information. Experiments were started on freshly purified samples, and each sample took between 9 and 10 days for completion. Combined application of these methods made it possible to assign essentially all residues in Switch I and Switch II for all K-Ras·GTP samples. The experimental temperature was kept at 298 K for the protein samples in complex with GDP, at 288 K for K-Ras(G12C)·GTP, and at 283 K for K-Ras(WT)·GTP and K-Ras(G12D)·GTP. To aid the transfer of the backbone NH assignments to room temperature, 3D HNCO experiments were repeated at 298 K on these GTP-bound samples. All the data were processed using NMRPipe^62^ and SMILE^63^ and visualized using NMRViewJ^64^, both via NMRBox^65^. Secondary structure propensity calculations for the three variants in their GDP-bound and GTP-bound forms were performed using the program SSP^20^ and TALOS-N^21^.

### Relaxation dispersion experiments and nanoparticle-assisted relaxation

Backbone amide ^15^N and ^1^H^N^ CPMG NMR relaxation dispersion experiments at 298 K were performed on 850 and 600 MHz NMR instruments, and amide ^15^N CEST^18^ experiments were performed for all samples on the 850 MHz instrument using a CEST mixing time of 150 ms and *B*_1_ field strengths as listed in Supplementary Table 1. All dynamics experiments were performed on freshly purified K-Ras·GTP samples and used for no more than 3 days before being replaced with a sample from the same batch and identical buffer that had been kept at 4 °C. CPMG and CEST profiles were analyzed collectively using ChemEx^18^, and all three GTP-bound variants were fitted to a model of two-site exchange. Bootstrap analyses were performed to determine the experimental errors in the fitted parameters. For interpretation of the results, random coil chemical shifts were predicted from the amino acid sequences of Switch I and Switch II using the POTENCI software^19^.

For all NASR experiments, Levasil CS40-120 colloidal anionic silica nanoparticles with an average diameter of 20 nm (ref. ^66^; obtained from Nouryon) were dialyzed and directly mixed into the protein-containing buffer. The final concentrations of silica nanoparticles in the NMR samples were between 0.5 and 1.5 μM. Backbone amide ^15^N *R*_1_ and *R*_2_ spin relaxation rates for samples in the absence and presence of silica nanoparticles were measured with an NMR magnetic field strength of 850 MHz using standard ^15^N *R*_1_ and *R*_1ρ_ relaxation experiments^67,68^ as described previously^16^ and analyzed as described in the Supplementary Information.

### Reporting summary

Further information on research design is available in the Nature Portfolio Reporting Summary linked to this article.

## Online content

Any methods, additional references, Nature Portfolio reporting summaries, source data, extended data, supplementary information, acknowledgements, peer review information; details of author contributions and competing interests; and statements of data and code availability are available at 10.1038/s41594-023-01070-z.

## Supplementary information

Supplementary Information Supplementary Figs. 1–6, Tables 1–3 and Discussion.

Reporting Summary

Supplementary Data 1 Data for supplementary figures.

## Source data

Source Data Fig. 2 Numerical source data.

Source Data Fig. 3 Numerical source data.

Source Data Fig. 4 Numerical source data.

Source Data Fig. 6 Numerical source data.

## Data Availability

NMR backbone resonance assignments for K-Ras·GTP WT, G12D and G12C have been deposited in the publicly accessible BMRB database (https://bmrb.io/) under accession codes 52021, 52023 and 52024. All relaxation dispersion, CEST and NASR results can be accessed at 10.5061/dryad.j6q573nm0. Source data are provided with this paper.
