# Supplementary material for: Excited-state observation of active K-Ras reveals differential structural dynamics of wild-type versus oncogenic G12D and G12C mutants
Source: Nat Struct Mol Biol. 2023 Aug 28;30(10):1446–55. doi: 10.1038/s41594-023-01070-z (PMC10584678; doi:10.1038/s41594-023-01070-z)
Supplement: Supplementary file 1 — Supplementary Figs. 1–6, Tables 1–3 and Discussion. [file 41594_2023_1070_MOESM1_ESM.pdf]

nature structural & molecular biology

Article https://doi.org/10.1038/s41594-023-01070-z

Excited-state observation of active K-Ras reveals differential structural dynamics of wild-type versus oncogenic G12D and G12C mutants

In the format provided by the authors and unedited

Table of Contents

A. NMR Resonance Assignment Experiments

B. NMR Relaxation and CEST Experiments

C. Standard NMR S^2 Model-Free Analysis vs. NASR

D. X-ray Structure Based Ensemble Construction and S^2 Back-Calculation

References

A. NMR Resonance Assignment Experiments

NMR spectra for the sequence-specific NMR resonance assignments were recorded on a Bruker Avance III 850 MHz spectrometer (Bruker, Billerica, MA), equipped with a 5 mm TCI triple resonance HCN cryoprobe and Z-axis gradient. A series of triple-resonance experiments (Sattler et al., 1999) was subsequently recorded using sensitivity-enhanced gradient coherence selection[1,2], semi-constant time acquisition in the ^{15}N dimension[3], and non-uniform sampling (NUS) following a Poisson-gap sampling schedule[4]. The direct ^{1}H dimension contained 2048 complex points with 86 ms acquisition time, while the typical acquisition times in the indirect dimensions (^{15}N/^{13}C), the number of complex points (*), and sparsity levels were as follows: HNCO : 50 ms / 25 ms (302* x 138* ; 2.6%); HN(CA)CO : 50 ms / 25 ms (302* x 138* ; 3.8%); HNCA : 40 ms / 14 ms (240* x 180* ; 3.9%); HN(CO)CA : 50 ms / 14 ms (302* x 180* ; 1.6%); HNCACB : 40 ms / 13.4 ms (240* x 460* ; 2.9%), and CBCA(CO)NH : 50 ms / 6.4 ms (300* x 220* ; 2.4%). Additionally, 3D ^{15}N-edited NOESY and 3D CNH-NOESY[5] were recorded using uniform sampling with a mixing time of 180 ms and acquisition times (number of complex points) of 6.6 ms ^{1}H (180*) x 10 ms ^{15}N (60*) x 86 ms ^{1}H (2048*) and 3.4 ms ^{13}C (116*) x 10.6 ms ^{15}N (64*) x 86 ms ^{1}H (2048*), respectively. Each sample took about 9-10 days to complete all experiments were performed on freshly purified, prepared samples. The coordination of the samples and the application of modern techniques made it possible to assign most of the residues in Switch I and Switch II for all K-Ras·GTP samples. The experimental temperature was kept at 298 K for the protein samples in complex with GDP, at 288 K for K-Ras(G12C)·GTP, and at 283 K for K-Ras(WT)·GTP and K-Ras(G12D)·GTP. To aid the transfer of the backbone NH assignments to room temperature, 3D HNCO was then repeated at 298 K on these GTP-bound samples. All the data were processed using NMRPipe[6]/SMILE[7] and visualized using NMRViewJ[8] both via NMRBox[9].

These experiments, along with the optimized NMR samples and experimental conditions, allowed the detection and assignment of essentially all backbone chemical shifts of both Switch I and II for K-Ras·GTP WT, G12D, and G12C. Specifically, 100% of the non-proline residues could be assigned for G12D and 98% for WT (missing assignments: Y64, S65, M72) and G12C (missing assignments: Q61, Y64, M72). Due to strong overlap of some residues in the 2D ^{15}N-^{1}H HSQC spectra, which serve as footprints of the pseudo-3D CPMG, CEST, and NASR dynamics experiments used in this work, a few residues were not amenable to fully quantitative dynamics

analysis (**Table S2**). In addition, two residues (D33, T35) for WT and four residues (I21, D30, D33, I36) for G12D mutant were too weak for quantitative NASR analysis as the presence of silica nanoparticles introduces additional line broadening preventing the determination of transverse ^{15}N-R_2 relaxation rates with high accuracy.

B. NMR Relaxation and CEST Experiments

All dynamics experiments were performed on freshly purified K-Ras·GTP samples and used for no more than 3 days before being replaced with a sample from the same batch and identical buffer that had been kept at 4°C. NMR relaxation dispersion experiments were acquired on an 850 MHz Bruker magnet equipped with a 5 mm TCI cryoprobe and a 600 MHz Bruker magnet equipped with a 5 mm TXI cryoprobe at 298 K. Amide ^{15}N CPMG experiments were acquired at both magnetic fields using either the CW-CPMG[10] or STCW-CPMG[11] pulse sequences. The constant relaxation time was set to 40 ms and the CPMG pulsing frequency, ν_{CPMG}, was varied from 25 Hz to 2 kHz on the 850 MHz instrument, and from 25 Hz to 1 kHz on the 600 MHz instrument. Amide ^{1}H^{N} CPMG experiments were acquired using the sequence of Yuwen and Kay[12]. A constant relaxation time of 16 ms was used and ν_{CPMG} was varied from 62.5 Hz to 4 kHz at the 850 MHz instrument only. Amide ^{15}N CEST[13] experiments were performed for all samples on the 850 MHz instrument using a CEST mixing time of 150 ms and B_1 field strengths as listed in **Table S1**. Additional amide HSQC/HMQC experiments were acquired at both 600 and 850 MHz to aid in the sign determination of small ^{15}N CPMG-derived chemical shift differences improving the robustness of the global fitting procedure[14,15].

NMR data was processed using nmrPipe[6] and intensities were extracted using either the autoFit routine in the NMRPipe suite or Voigt fitter[16]. Profiles were analyzed collectively using ChemEx[13]. Errors in signal amplitudes were estimated from 2-3 replicate ν_{CPMG} measurements for each CPMG experiment and used to propagate the errors in $R_{2,\mathrm{eff}}$, where

$$R_{2,\mathrm{eff}} = -\frac{\ln\left[\frac{I(\nu_{\mathrm{CPMG}})}{I_0}\right]}{T_{\mathrm{rlx}}}$$

I_0 is the signal amplitude without the CPMG element, $I(\nu_{\mathrm{CPMG}})$ is the amplitude at the given ν_{CPMG}, and T_{rlx} is the constant relaxation time. The median error in $R_{2,\mathrm{eff}}$ was 0.4 s^{-1} for ^{15}N datasets and 0.8 s^{-1} for ^{1}H^{N} datasets, however the error in each measurement varies as it is dependent on the signal amplitude. The ^{15}N and ^{1}H^{N} CPMG profiles were first analyzed to identify those residues with $R_{\mathrm{ex}} = R_{2,\mathrm{eff}}(\min(\nu_{\mathrm{CPMG}})) - R_{2,\mathrm{eff}}(\max(\nu_{\mathrm{CPMG}}))$ greater than 1.65 times the R_{ex} measurement error, $\sigma_{R\mathrm{ex}}$, amounting to 95% confidence for the presence of exchange. Those residues identified in this fashion were then fit to simple analytical models of fast and slow exchange[17] to estimate the initial 2-state exchange parameters, the total rate $k_{\mathrm{ex}} = k_{21} + k_{12}$ and population p_1, serving as

input for subsequent global analysis. In the presence of GTP, all three K-Ras variants examined here showed significant dispersions with R_{ex} values up to 40 s^{-1}. Using the 2-state relationship[18] $R_{ex} \approx \frac{p_2 p_1 \Delta\omega^2 k_{ex}}{p_2 \Delta\omega^2 + k_{ex}^2}$ (where $p_2 = 1 - p_1$ and $\Delta\omega$ is the chemical shift difference between the ground and the excited state in units of rad/s) dispersions in the intermediate-to-slow regime with values of $\frac{|\Delta\omega|}{k_{ex}} > 0.5$ are required to define exchange parameters accurately, whereby a R_{ex} cutoff of 5 s^{-1} was chosen to select residues for inclusion in subsequent global analysis. Global analysis of these residues using all datasets simultaneously via numerical simulation of the pulse sequences was performed with ChemEx, thus circumventing any requirement for model selection. The selected residues fit well to a 2-site exchange process, and ^{15}N CEST showed no evidence of additional states, therefore exchange models with more than 2 states were not pursued. Errors in the global exchange parameters were determined from 1000 iterations of a bootstrap analysis of the selected data. With the global parameters defined, a final fit was performed with k_{ex} and p_1 fixed in order to determine $\Delta\varpi$ for all residues. Sign information was obtained from ^{15}N CEST and/or HSQC/HMQC measurements where available. A final bootstrap analysis of the data was performed to determine the errors in $\Delta\varpi$ (ppm).

Nanoparticle-assisted Spin Relaxation (NASR) Experiments

For all nanoparticle-assisted spin relaxation experiments (NASR), Levasil CS40-120 colloidal anionic silica nanoparticles (SNPs) with an average diameter of 20 nm[19] (obtained from Nouryon™) were dialyzed and directly mixed into the protein-containing buffer. The final concentrations of SNPs in the samples were between 0.5 and 1.5 µM. Backbone amide ^{15}N R_1 and R_2 spin relaxation rates for samples both in the absence and presence of SNPs were measured at 850 MHz NMR magnetic field strength using standard ^{15}N R_1 and $R_{1\rho}$ relaxation experiments[20,21] as described previously[22]. The recovery delays were set to 2 s, and the $R_{1\rho}$ spinlock field strength was around 2000 Hz. Peak fitting errors were estimated from replicate delays in the measurements and propagated by analytical error propagation or Monte Carlo simulations through the analysis. The enhancements ΔR_2 of the ^{15}N transverse relaxation R_2 rates upon addition of SNPs, which can be expressed as[22]

$$\Delta R_2 = R_{2,NP} - R_{2,free}$$

were converted to NASR order parameters S^2(NASR) by global scaling according to

$$S^2 = \Delta R_2/(c\, \tau_{NP} p_{bound})$$

whereby τ_{NP} is the tumbling correlation time of the nanoparticles and p_{bound} is the nanoparticle-bound protein population. The global scaling factor c was determined using residues that are part of rigid secondary structures so that their S^2(NASR) matched the model-free S^2(MF) ≈ 0.88 (see **Figure S5**).

C. Standard NMR S^2 Model-Free Analysis vs. NASR

To compare the NASR results with standard NMR N-H S^2 order parameters of K-Ras in the absence of SNPs, the lean version of the model-free (LMF) approach based on ^{15}N R_1 and R_2 relaxation rates was used using the following spectral density function[23]

$$J(\omega) = S^2 \frac{\tau_P}{1+\omega^2\tau_P^2} + (1-S^2)\frac{\tau_{i,P}}{1+\omega^2\tau_{i,P}^2}, \quad \tau_{i,P}^{-1} = \tau_i^{-1} + \tau_P^{-1}$$

where τ_i is a residue-specific internal correlation time and τ_P is the protein rotational correlation time determined from the R_1/R_2 ratios of rigid regions with well-defined secondary structures. At 298 K τ_P is around 10 ns for K-Ras. For model-free analysis, the N-H bond length r_{NH} was set to 1.02 Å and the ^{15}N chemical shift anisotropy $\Delta\sigma$ was set to -172 ppm.

Standard model-free (MF) analysis reports about internal motions on the low ns and sub-ns timescale, because it is insensitive to dynamics processes that are comparable or slower than the rotation tumbling correlation time τ_P of the free protein in solution, which is about 10 ns for K-Ras at 298 K. The comparison between S^2(NASR) and S^2(MF) is shown for K-Ras·GDP and K-Ras·GTP in **Figure S5**. It shows how NASR is sensitive to large scale motions in K-Ras·GDP in Switch I and II that are undetectable by MF suggesting that these motions occur on 10 ns to 1 μs timescales. By contrast, the backbone dynamics of K-Ras·GTP are much more restrained taking place on the sub-ns timescale window for which MF and NASR yield essentially equivalent results.

D. X-ray Structure Based Ensemble Construction and S^2 Back-Calculation

The K-Ras·GDP X-ray crystal structures in the Mg^{2+}-bound apo state (without other ligands or binding partners present) reviewed in Ref. [24] served as the pool for the structure selection and minimal ensemble construction shown in **Figure 6**. The hydrogen atom coordinates were added using UCSF ChimeraX[25] for crystal structures without hydrogen atoms present. The structures were visualized using PyMOL and aligned with respect to all heavy atoms of the residues that are *not* in the P-loop (10-17), Switch I (30-38), or Switch II (60-76) regions through the extra fit align method in PyMOL. The crystal structure figures were generated with PyMOL.

For an ensemble with multiple conformational states, the backbone N-H S^2 order parameter of each residue can be calculated using equation $S^2 = \sum_{i,j} p_i p_j P_2(cos\theta_{ij})$, where p_i, p_j are the populations of states i, j in the ensemble, θ_{ij} is the angle between the N-H bond vectors of states i and j, and $P_2(x) = (1/2)(3cos^2x - 1)$ is the second-order Legendre polynomial[26]. The obtained S^2 were globally scaled by 0.88 to account for omnipresent fast local fluctuations so that the back-calculated and experimental S^2 values of residues with rigid secondary structures are matched. The population of each state (X-ray structure) in the ensemble was determined by minimizing the squared mean deviations between ensemble-derived and experimental S^2 values of residues that are in the Switch I and Switch II regions.

The minimal ensemble that can best reproduce the S^2(NASR) profile consists of a WT structure (PDB entry 6MBU), a G12D mutant structure 4EPR, and an A59G mutant structure 6ASE with populations of 47%, 39%, and 14%, respectively (**Figure 6** in the main text). Adding one more WT structure (PDB entry 4OBE) to this minimal ensemble does not further improve the agreement with the experiment. The optimized fitted populations are 4% (4OBE), 48% (6MBU), 35% (4EPR), and 13% (6ASE). The low population of 4OBE and the shallowness of the χ^2 surface for the 4OBE population close to zero, as shown in **Figure S6F**, indicates that inclusion of 4OBE has a minimal effect on the agreement with the experimental S^2(NASR) profile. The RMSDs between the back-calculated S^2 and WT K-Ras·GDP S^2(NASR) values are 0.11 for the Switch I region and 0.06 for Switch II, which are similar to the RMSDs of the minimal ensemble in **Figure 6** (0.12 for Switch I and 0.06 for Switch II). This is because 4OBE adopts a Switch I conformation that is essentially identical to that of 6MBU and 4EPR, while the orientation of its Switch II-α2 helix is nearly parallel with that of 6ASE (**Figure S6G**).

The existing WT and G12D K-Ras·GDP crystal structures have Switch II regions that are sufficiently diverse to explain the S^2(NASR) profile, as demonstrated in **Figure S6D**. An ensemble consisting of three WT crystal structures (PDB entries 4OBE, 6MBU, and 5W22 (5W22 has the engineered mutation C118S)) and a G12D mutant structure (PDB entry 4EPR, which also contains the C118S mutation) with populations of 9% (4OBE), 22% (6MBU), 23% (5W22), and 46% (4EPR) can reproduce the S^2(NASR) profile of the Switch II region. However, these structures adopt nearly identical Switch I conformations and thus cannot explain the S^2(NASR) dip in the Switch I region.

Table S1. Summary of acquired relaxation dispersion data. For ^{15}N CPMG, the pulse sequence used for acquisition is indicated. For ^{15}N CEST, the calibrated B_1-field strengths and saturation times are provided.

Sample		^{15}N CPMG	$^1H^N$ CPMG	HSQC/HMQC	^{15}N CEST
**GTP**	**WT**	STCW	X	X	28 Hz / 150 ms
	**G12C**	CW			43 Hz / 150 ms
	**G12D**	STCW	X	X	44 Hz / 150 ms
**GDP**	**WT**	STCW			42 Hz / 150 ms
	**G12C**	CW			44 Hz / 150 ms
	**G12D**	CW & STCW			45 Hz / 150 ms

Table S2. Assignment summary and number of quantifiable peaks in amide ^{15}N relaxation and dispersion datasets. K-Ras includes 169 residues plus an N-terminal Ser-Asn-Ala tag from purification. The first observable residue is the Alanine in the tag, residue 0, with the remaining residues numbering 1-169, which follows convention. Residues 34, 110, 121, and 140 are prolines and do not appear in amide ^{15}N experiments.

Sample		UNASSIGNED	QUANTIFIABLE W/O SNP	QUANTIFIABLE WITH SNP
GDP	WT	None	160 / 166 (96.4%)	158 / 166 (95.2%)
	G12C	None	161 / 166 (97.0%)	159 / 166 (95.8%)
	G12D	None	161 / 166 (97.0%)	160 / 166 (96.4%)
GTP	WT	Y64, S65, M72	157 / 166 (94.5%)	155 / 166 (93.4%)
	G12C	Q61, Y64, M72	152 / 166 (91.5%)	152 / 166 (91.5%)
	G12D	None	154 / 166 (92.8%)	150 / 166 (90.4%)

Table S3. Oligonucleotide sequences.

Oligonucleotide	Sequence
K-Ras wt forward	$^{5'}$TACTTCCAATCCAATGCAATGACTGAATATAAACTTGTGGTAGTTGG AGCTGGTGGC$^{3'}$
K-Ras wt reverse	$^{5'}$TTATCCACTTCCAATGTCACTTTTCTTTATGTTTTCGAATTTCTCGAA CTAATGTATAGA$^{3'}$
K-Ras G12C mutagenesis forward	$^{5'}$GTGGTAGTTGGAGCTTGTGGCGTAGGC$^{3'}$
K-Ras G12C mutagenesis reverse	$^{5'}$TCTTGCGTACGCCACAAGCTCCAACTA$^{3'}$
K-Ras G12D mutagenesis forward	$^{5'}$TGGTAGTTGGAGCTGATGGCGTAGGCA$^{3'}$
K-Ras G12D mutagenesis reverse	$^{5'}$CTCTTGCCTACGCCATCAGCTCCAACT$^{3'}$

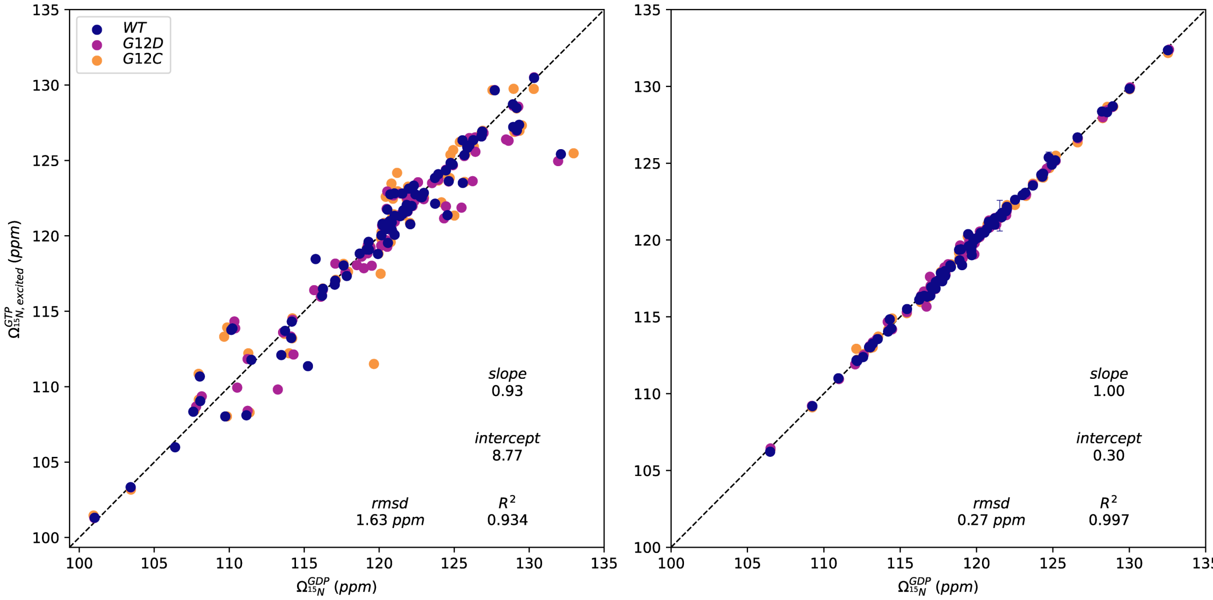

Figure S1. Correlation of excited state shifts of K-Ras·GTP with the chemical shifts of K-Ras·GDP. On the left are residues from the N-terminal effector lobe (residues 1-86), while on the right is the remainder of the residues (87-169) of the C-terminal allosteric lobe.

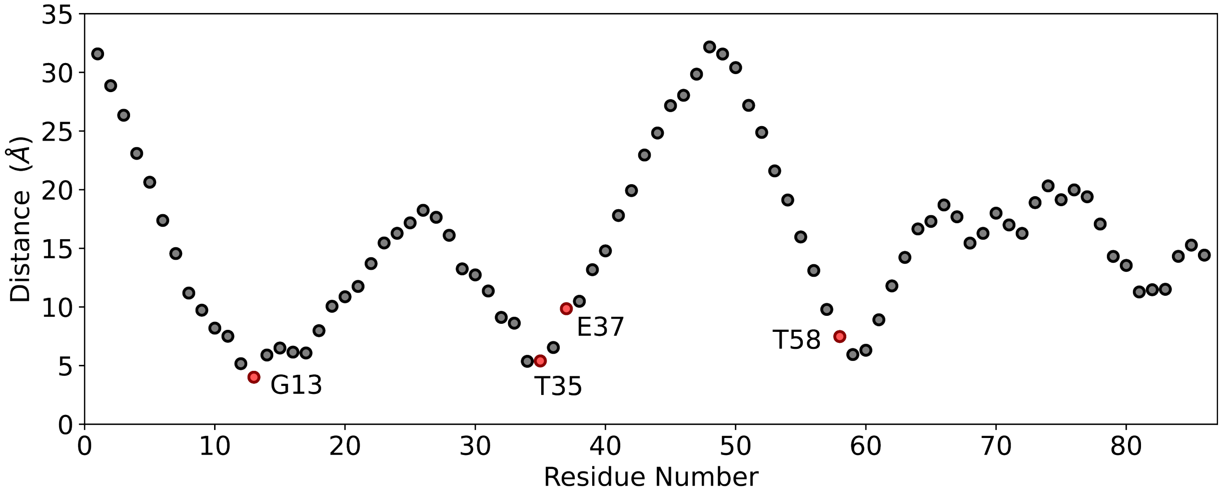

Figure S2. The proximity of backbone nitrogen atoms in K-Ras to the γ-phosphate of GTP according to X-ray crystal structures. The distances were calculated using the crystal structures of WT K-Ras·GDP (PDB entry 4OBE), K-Ras·GTP (PDB entry 5VQ2), and K-Ras·GTPγS (PDB entry 5VQ6). Structures 4OBE and 5VQ6 were aligned with respect to 5VQ2, and the γ-phosphate position was taken from 5VQ2. The distances calculated from those three structures were then averaged. Residues G13, T35, E37, and T58, which exhibit the largest deviations from the diagonal in **Figure 3G**, are indicated in red. It suggests that deviations from the diagonal in **Figure 3G** are a consequence of the difference in ligand chemistry between the excited state of K-Ras·GTP and K-Ras·GDP, rather than structural dynamics. Residues 87-169 are more than 12 Å away from the γ-phosphate and are not shown.

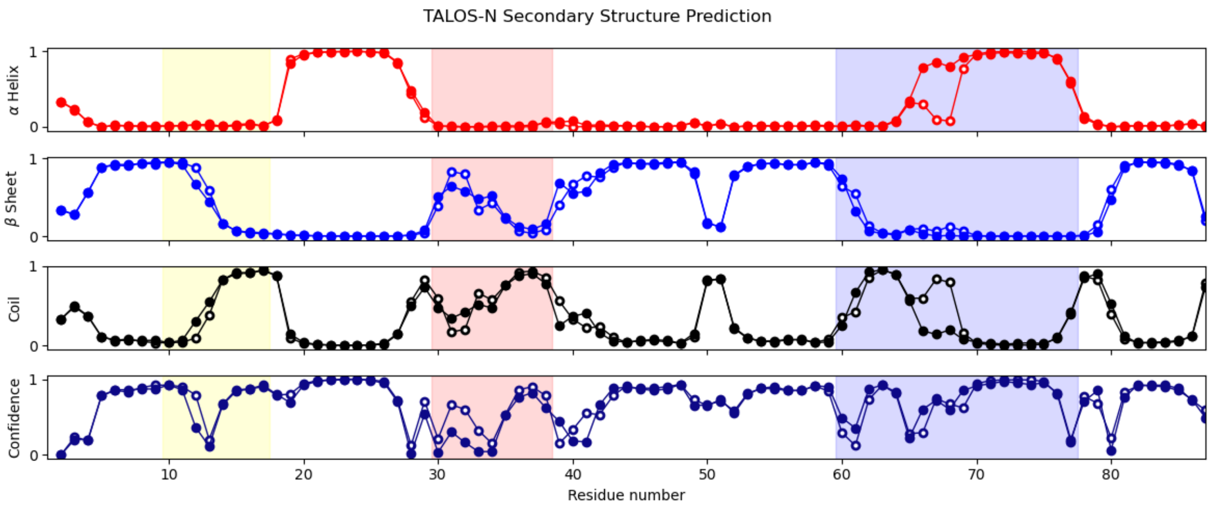

Figure S3. TALOS-N[27] prediction of secondary structure based on assigned NMR chemical shifts. Filled symbols represent WT K-Ras·GTP while open symbols represent WT K-Ras·GDP. P-Loop, Switch I, and Switch II are highlighted in yellow, red, and blue, respectively. The largest differences occur in the first half of Switch II, followed by the first half of Switch I and the P-Loop, which is consistent with the results obtained from SSP analysis (**Figure 4** in the main text).

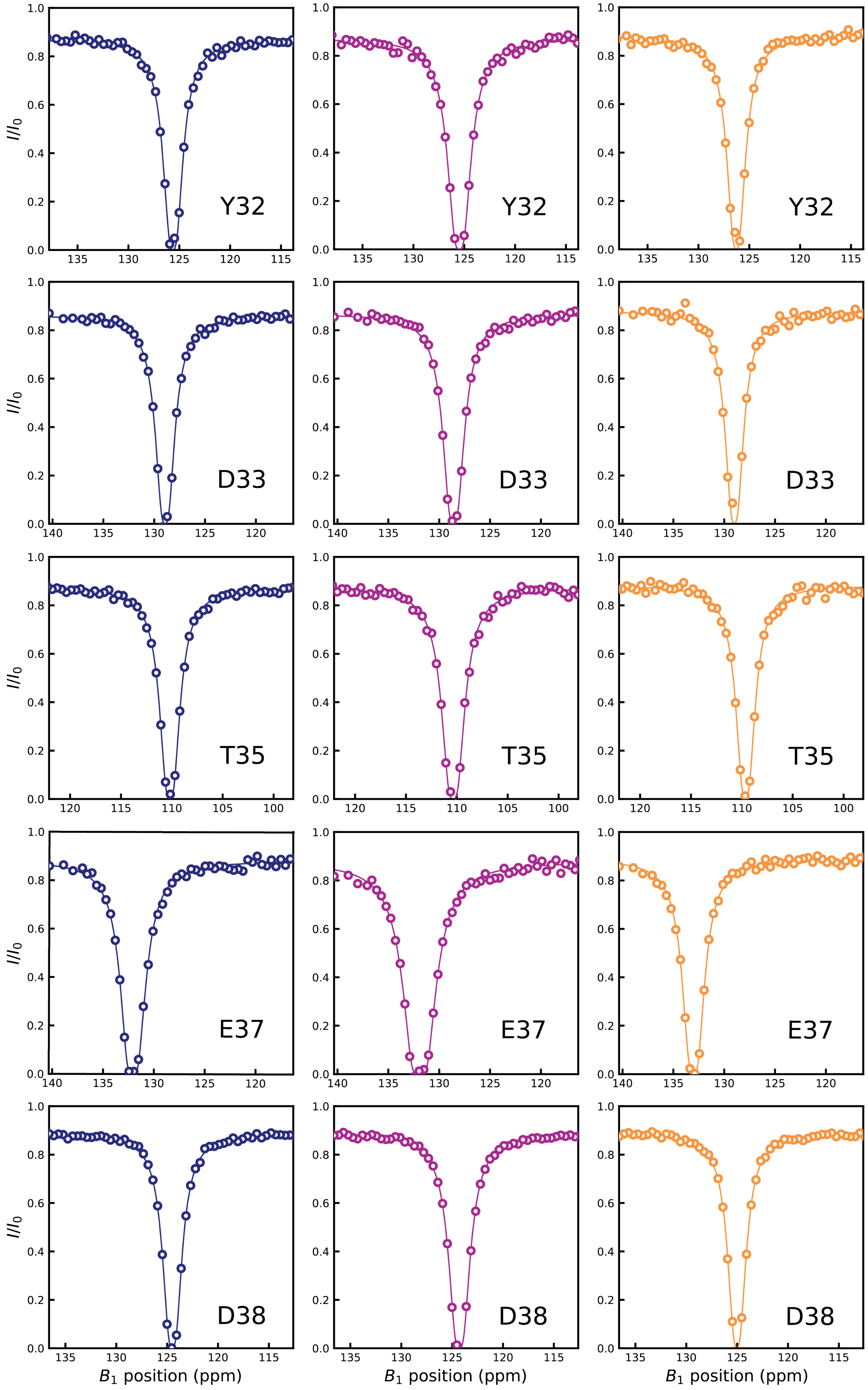

Y32
Y32
Y32
D33
D33
D33
T35
T35
T35
E37
E37
E37
D38
D38
D38
I/I_0
B_1 position (ppm)

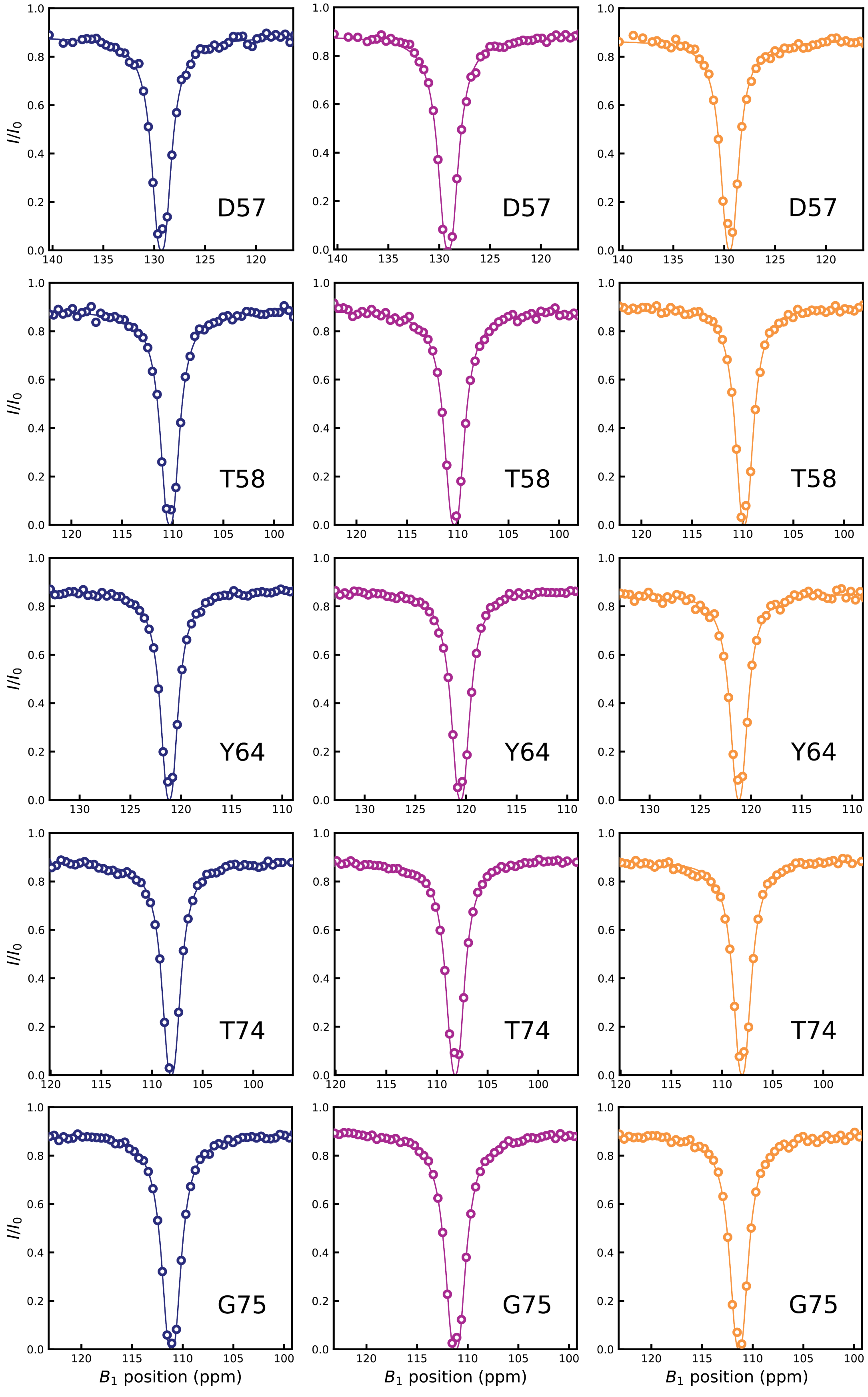
D57
T58
Y64
T74
G75
I/I_0
B_1 position (ppm)

Figure S4. Illustration of experimental ^{15}N CEST profiles observed in WT K-Ras·GDP and the G12D and G12C mutants in dark blue, purple, and orange respectively. Residues are included from Switch I (Y32, D33, T35, E37, and D38) and Switch II (D57, T58, Y64, T74, and G75) and show no evidence of secondary states probed on the millisecond timescale. Data were acquired at 850 MHz NMR field strength with B_1 fields specified in Table S1.

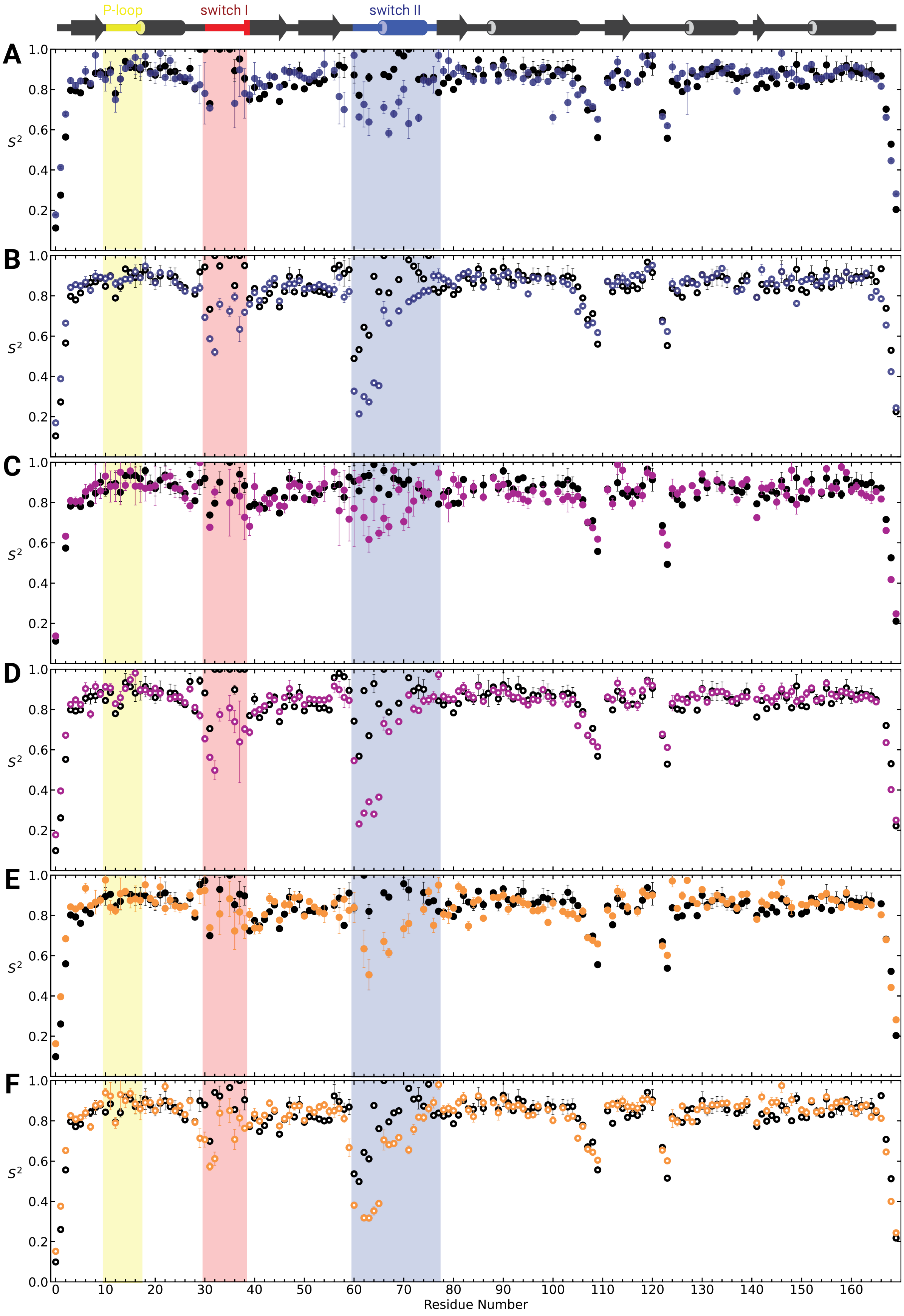

P-loop
switch I
switch II
A
B
C
D
E
F
S^2
Residue Number

Figure S5. Comparison between backbone dynamics of K-Ras·GTP (filled circles) and K-Ras·GDP (open circles) by NASR and model-free (MF) analysis. Panels A-F show the backbone N-H S^2 order parameters derived from the traditional model-free analysis (black) and NASR (WT: dark blue, G12D: purple, and G12C: orange) of **A**) WT K-Ras·GTP, **B**) WT K-Ras·GDP, **C**) G12D K-Ras·GTP, **D**) G12D K-Ras·GDP, **E**) G12C K-Ras·GTP, and **F**) G12C K-Ras·GDP. The P-loop, Switch I, and Switch II regions are highlighted with yellow, red, and blue shadings, respectively. The much lower values of S^2(NASR) than S^2(MF) in the Switch II region of K-Ras·GDP suggest the presence of large amplitude ns-μs motions in this region. Values and error bars are as described in the main text **Figure 4**.

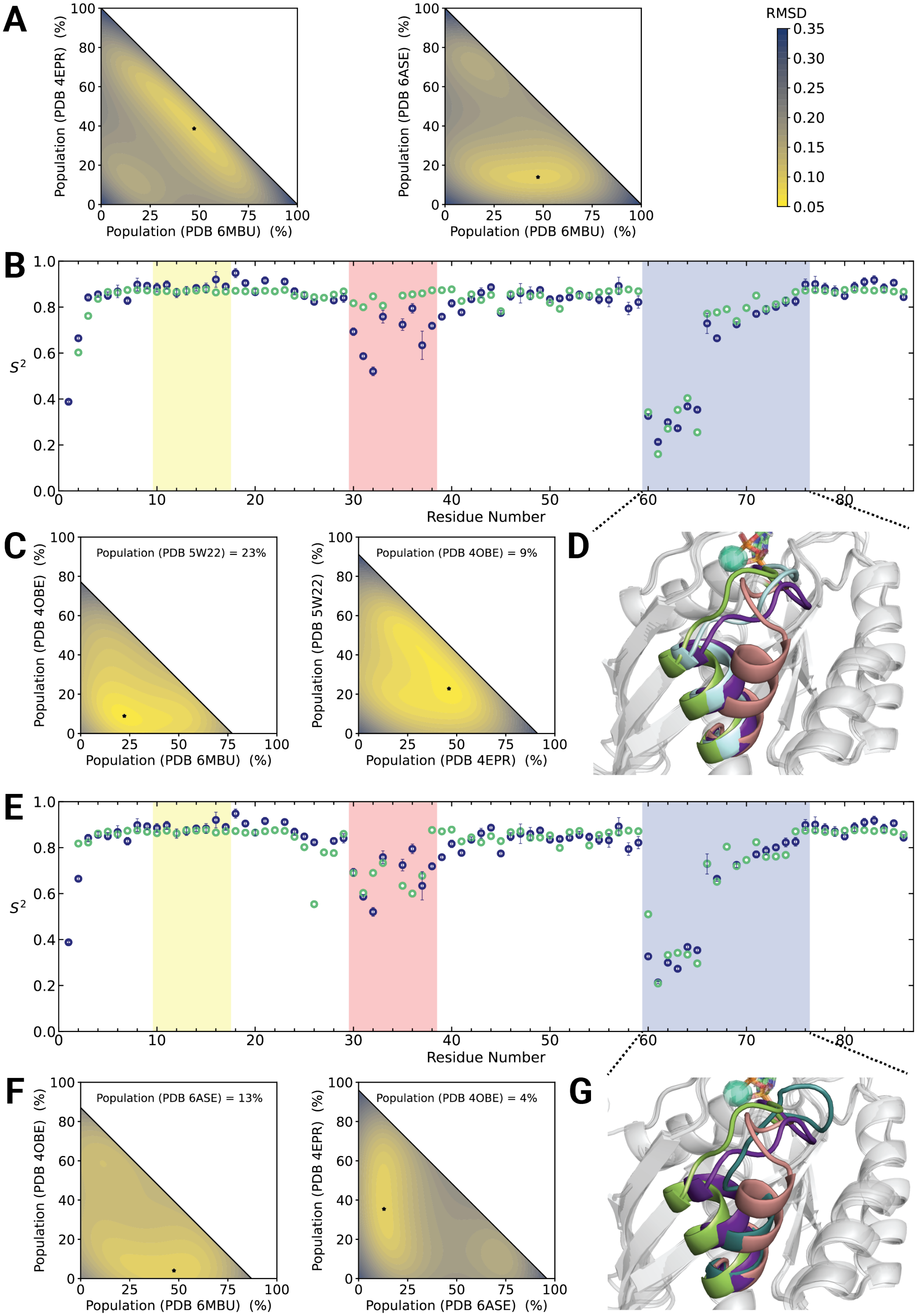
A
B
C
D
E
F
G
RMSD
Population (PDB 6MBU) (%)
Population (PDB 4EPR) (%)
Population (PDB 6ASE) (%)
Population (PDB 4OBE) (%)
Population (PDB 5W22) (%)
Population (PDB 5W22) = 23%
Population (PDB 4OBE) = 9%
Population (PDB 6ASE) = 13%
Population (PDB 4OBE) = 4%
S^2
Residue Number

Figure S6. X-ray-structure derived ensemble of K-Ras·GDP Switch regions, back-calculated backbone N-H order parameters, and the χ^2 surfaces for the population determinations. **A**) The changes of χ^2 (the RMSD between the back-calculated S^2 and experimental S^2(NASR) values of the Switch I and Switch II regions) as a function of the populations of the structures of the minimal ensemble of **Figure 6** in the main text. The black star indicates the global minimum. **B-D**) The ensemble consisting of three WT crystal structures (PDB entries 4OBE, 6MBU, and 5W22) and a G12D mutant structure (PDB entry 4EPR). Populations of 9% (4OBE), 22% (6MBU), 23% (5W22), and 46% (4EPR) can best reproduce the S^2(NASR) profile of the Switch II region. **B**) Back-calculated S^2 values (green) in comparison with the WT K-Ras·GDP S^2(NASR) profile (dark blue). The S^2(NASR) data are presented as the best fit ± one standard deviation, as described in the caption of **Figure 4**. **C**) Representative 2D cross-sections of the χ^2 surface (RMSD of the Switch II region). **D**) The Switch II region structures with the following coloring: 4OBE (salmon), 6MBU (green), 5W22 (light blue), and 4EPR (dark purple). **E-G**) The ensemble after adding one more WT structure (PDB entry 4OBE) to the minimal ensemble in **Figure 6**. Fitting gives optimized populations of 4% (4OBE, WT), 48% (6MBU, WT), 35% (4EPR, G12D), and 13% (6ASE, A59G), with **E**) back-calculated S^2 values (green) and **F**) representative 2D cross-sections of the χ^2 surface (RMSD of the Switch I and Switch II regions). **G**) The Switch II conformations of 4OBE (salmon), 6MBU (green), 4EPR (dark purple), and 6ASE (dark cyan).

The yellow, red, and blue shadings in Panels B and E highlight the P-loop, Switch I, and Switch II, respectively. The color-coding of Panels C and F is the same as in Panel A. In Panels D and G, Mg^{2+} ions are shown as teal spheres and GDP in stick representation. The experimental WT K-Ras·GDP S^2(NASR) profiles (dark blue) in Panels B and E are the same as the ones in **Figure 4B** in the main text.

References

1. Palmer, A. G., Cavanagh, J., Wright, P. E. & Rance, M. Sensitivity improvement in proton-detected two-dimensional heteronuclear correlation NMR spectroscopy. *J. Magn. Reson.* **93**, 151–170 (1991).

2. Schleucher, J. *et al.* A general enhancement scheme in heteronuclear multidimensional NMR employing pulsed field gradients. *J. Biomol. NMR* **4**, 301–306 (1994).

3. Grzesiek, S. & Bax, A. Amino acid type determination in the sequential assignment procedure of uniformly 13C/15N-enriched proteins. *J. Biomol. NMR 1993 32* **3**, 185–204 (1993).

4. Hyberts, S. G., Takeuchi, K. & Wagner, G. Poisson-gap sampling and forward maximum entropy reconstruction for enhancing the resolution and sensitivity of protein NMR data. *J. Am. Chem. Soc.* **132**, 2145–2147 (2010).

5. Diercks, T., Coles, M. & Kessler, H. An efficient strategy for assignment of cross-peaks in 3D heteronuclear NOESY experiments. *J. Biomol. NMR 1999 152* **15**, 177–180 (1999).

6. Delaglio, F. *et al.* NMRPipe: a multidimensional spectral processing system based on UNIX pipes. *J. Biomol. NMR* **6**, 277–93 (1995).

7. Ying, J., Delaglio, F., Torchia, D. A. & Bax, A. Sparse multidimensional iterative lineshape-enhanced (SMILE) reconstruction of both non-uniformly sampled and conventional NMR data. *J. Biomol. NMR* **68**, 101–118 (2017).

8. Johnson, B. A. & Blevins, R. A. NMR View: A computer program for the visualization and analysis of NMR data. *J. Biomol. NMR 1994 45* **4**, 603–614 (1994).

9. Maciejewski, M. W. *et al.* NMRbox: A Resource for Biomolecular NMR Computation. *Biophys. J.* **112**, 1529–1534 (2017).

10. Hansen, D. F., Vallurupalli, P. & Kay, L. E. An improved 15N relaxation dispersion experiment for the measurement of millisecond time-scale dynamics in proteins. *J. Phys. Chem. B* **112**, 5898–904 (2008).

11. Jiang, B., Yu, B., Zhang, X., Liu, M. & Yang, D. A 15N CPMG relaxation dispersion experiment more resistant to resonance offset and pulse imperfection. *J. Magn. Reson.* **257**, 1–7 (2015).

12. Yuwen, T. & Kay, L. E. Revisiting 1HN CPMG relaxation dispersion experiments: a simple modification can eliminate large artifacts. *J. Biomol. NMR* **73**, 641–650 (2019).

13. Vallurupalli, P., Bouvignies, G. & Kay, L. E. Studying 'invisible' excited protein states in slow exchange with a major state conformation. *J. Am. Chem. Soc.* **134**, 8148–8161 (2012).

14. Skrynnikov, N. R., Dahlquist, F. W. & Kay, L. E. Reconstructing NMR spectra of 'invisible' excited protein states using HSQC and HMQC experiments. *J. Am. Chem. Soc.* **124**, 12352–60 (2002).

15. Vallurupalli, P., Bouvignies, G. & Kay, L. E. Increasing the Exchange Time-Scale That

Can Be Probed by CPMG Relaxation Dispersion NMR. *J. Phys. Chem. B* **115**, 14891–14900 (2011).

16. Li, D.-W. W., Leggett, A., Bruschweiler-Li, L. & Brüschweiler, R. COLMARq: A Web Server for 2D NMR Peak Picking and Quantitative Comparative Analysis of Cohorts of Metabolomics Samples. *Anal. Chem.* **94**, 8674–8682 (2022).
17. Palmer, A. G., Kroenke, C. D. & Loria, J. P. Nuclear magnetic resonance methods for quantifying microsecond-to-millisecond motions in biological macromolecules. *Methods Enzymol.* **339**, 204–38 (2001).
18. Ishima, R. & Torchia, D. A. Estimating the time scale of chemical exchange of proteins from measurements of transverse relaxation rates in solution. *J. Biomol. NMR* **14**, 369–72 (1999).
19. Zhang, B., Xie, M., Bruschweiler-Li, L., Bingol, K. & Brüschweiler, R. Use of Charged Nanoparticles in NMR-Based Metabolomics for Spectral Simplification and Improved Metabolite Identification. *Anal. Chem.* **87**, 7211–7217 (2015).
20. Lakomek, N. A., Ying, J. & Bax, A. Measurement of 15N relaxation rates in perdeuterated proteins by TROSY-based methods. *J. Biomol. NMR* **53**, 209–221 (2012).
21. Gairí, M. *et al.* An optimized method for 15N R1 relaxation rate measurements in non-deuterated proteins. *J. Biomol. NMR* **62**, 209–220 (2015).
22. Xie, M. *et al.* Functional protein dynamics on uncharted time scales detected by nanoparticle-assisted NMR spin relaxation. *Sci. Adv.* **5**, eaax5560 (2019).
23. Lipari, G. & Szabo, A. Model-free approach to the interpretation of nuclear magnetic resonance relaxation in macromolecules. 1. Theory and range of validity. *J. Am. Chem. Soc.* **104**, 4546–4559 (1982).
24. Pantsar, T. The current understanding of KRAS protein structure and dynamics. *Comput. Struct. Biotechnol. J.* **18**, 189–198 (2020).
25. Pettersen, E. F. *et al.* UCSF ChimeraX: Structure visualization for researchers, educators, and developers. *Protein Sci.* **30**, 70–82 (2021).
26. Brüschweiler, R. *et al.* Influence of Rapid Intramolecular Motion on NMR Cross-Relaxation Rates. A Molecular Dynamics Study of Antamanide in Solution. *J. Am. Chem. Soc.* **114**, 2289–2302 (1992).
27. Shen, Y. & Bax, A. Protein backbone and sidechain torsion angles predicted from NMR chemical shifts using artificial neural networks. *J. Biomol. NMR* **56**, 227–41 (2013).
